# Rapid unimolecular reactions of acyl peroxy radicals: extending the structure–activity relationships†

**DOI:** 10.1039/d5cp01175b

**Published:** 2025-06-03

**Authors:** Lauri Franzon, Anni Savolainen, Siddharth Iyer, Matti Rissanen, Theo Kurtén

**Affiliations:** a Department of Chemistry, University of Helsinki, P.O. Box 55 (A.I. Virtasen aukio 1) 00014 Helsinki Finland lauri.franzon@helsinki.fi theo.kurten@helsinki.fi; b Aerosol Physics Laboratory, Physics Unit, Tampere University 33720 Tampere Finland

## Abstract

Acyl peroxy radicals are especially efficient at forming organic accretion products in the troposphere, but they also have short lifetimes due to rapid unimolecular reactions. For this reason, we find it important to accurately represent the reactions of these species in structure–activity relationships estimating the unimolecular reactivity of atmospheric peroxy radicals. To address this, we performed multi-conformer transition state theory calculations to determine H-shift and ring closure reaction rates for aldehyde-substituted and unsaturated acyl peroxy radicals over a wide temperature range. Similar calculations were performed for enol-substituted peroxy radicals, which are also underrepresented in SAR models. As a results, we found that H-shifts from aldehyde groups are highly competitive, that ring closures are overwhelmingly the major atmospheric fate of unsaturated acyl peroxy radicals, and that H-shifts from *Z*-enols outcompete all other unimolecular and bimolecular reactions whenever they are possible. In conclusion, in extending the SAR models we have gained valuable insight on some of the most rapid reactions for any peroxy radicals in the atmosphere.

## Abstract 2

We have used computational methods to extend the structure–activity relationships for peroxy radical H-shifts and ring closure reactions, with a particular focus on the rapid reactions of multi-functional acyl peroxy radicals.

## Graphical abstract

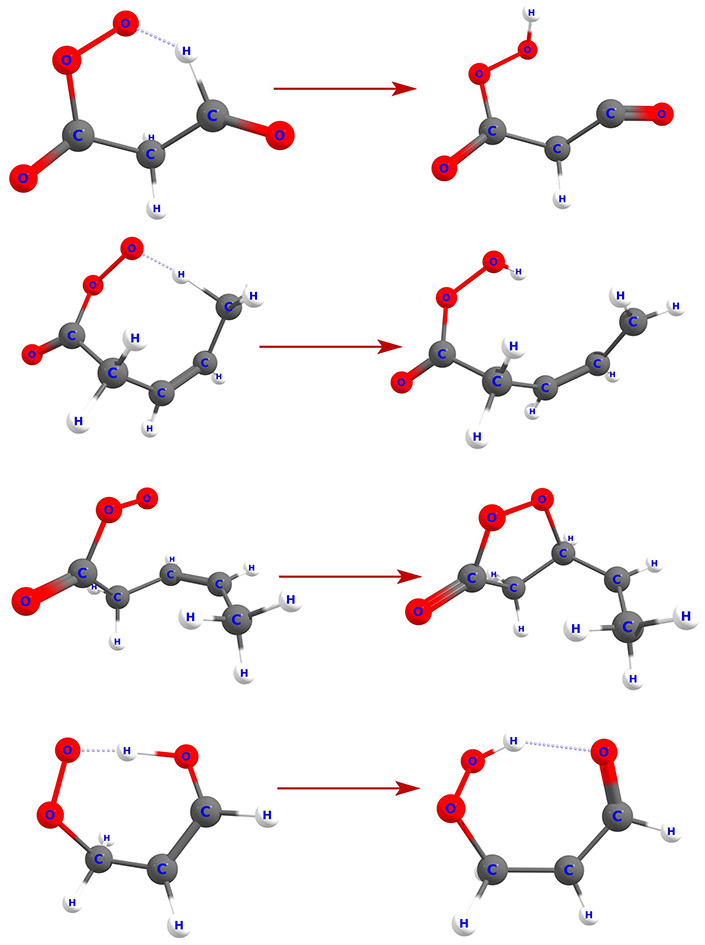

## 1 Introduction

Organic peroxy radicals (RO_2_) are all-important for the atmospheric oxidation of volatile organic compounds (VOC) for two reasons: firstly, they form in abundance in all atmospheric environments where organic vapours are present, due to O_2_ molecules adding to carbon-centred radicals. Secondly, the competition among the various reactions of RO_2_ are an important branching point for the subsequent chemistry, with important implications for the environmental impact. In polluted conditions, reactions between RO_2_ and NO and NO_3_ radicals largely form alkoxy radicals (RO) and nitrogen dioxide (NO_2_), which typically leads to fragmentation into smaller organic molecules for the former and photolysis for the latter, contributing to ozone (O_3_) pollution.^1^ In clean and remote environments, however, the impact of RO_2_ chemistry is dependent on the competition between two reactions. RO_2_ either forms closed-shell hydroperoxides (ROOH) through reactions with hydroperoxy radicals (HO_2_), or reacts with other RO_2_ in peroxy radical recombination (RO_2_ + RO_2_) reactions. The latter is a complex reaction with multiple channels which has received increasing attention in recent years due to being a major source of low-volatility organic accretion products which may condense on aerosol particle surfaces.^2^ Tropospheric daytime concentrations of HO_2_ are roughly equal to those of total RO_2_ on average,^3^ and as such the former is generally viewed to be the dominant reaction channel due to the latters high variability in reaction rates.^4^ It is however uncertain whether this is the case in all pristine environments, since we lack accurate estimates of the relative abundance of rapidly and slowly recombining RO_2_.^3^ The key to determining the relative importance of RO_2_ + RO_2_ reactions in clean environments is then to quantify the concentrations of RO_2_ species with systematically rapid RO_2_ + RO_2_ rates.

The most notable RO_2_ species with systematically rapid bimolecular reaction rates are acyl peroxy radicals (RC(O)O_2_), which form from O_2_ addition to acyl radicals (RCO).^5^ While their rate coefficients for reactions with NO_*x*_ and HO_2_ are also higher than for non-acyl RO_2_, their collision-limited RO_2_ + RO_2_ reactions are especially notable, as this allows them to undergo rapid cross reactions with RO_2_ species for which both self-reactions and other RO_2_ + RO_2_ cross reactions are systematically slow.^4,6,7^ Unlike typical RO_2_, RC(O)O_2_ also have non-negligible reaction rates for bimolecular addition to alkenes,^8^ and this has recently been demonstrated to form stable accretion products in laboratory conditions.^9^ Both of these observations underline the importance of constraining the distribution of ambient RC(O)O_2_ species for the study of gas-phase formation of organic accretion products. The most common RC(O)O_2_ compound, the acetyl peroxy radical (CH_3_C(O)O_2_), has been shown to readily partake in a variety of RO_2_ + RO_2_ reactions,^6^ but similar data is scarcer for more complex RC(O)O_2_ compounds. A main reason for this is that these more complex RC(O)O_2_ are known to have rapid unimolecular hydrogen shift (H-shift) reactions resulting in either carbon-centered radicals or isomeric RO_2_, in case the H-shift occurred from a hydroperoxide group. In the former case the reaction is often immediately followed by O_2_ addition to the carbon-centered radical, in which case the process is known as autoxidation.^10–12^ The latter type of H-shift is better known as H-scrambling, which is considered rapid and reversible for all RO_2_ except RC(O)O_2_.^13^ A recent computational study by Seal *et al.*^14^ evaluated H-shift reaction rates for larger RC(O)O_2_, but only for linear, unsubstituted RC(O)O_2_. The highly systematic structure–activity relationship (SAR) for H-shift reactions of general RO_2_ by Vereecken & Nozière (from now on H-SAR)^15^ suggest that the fastest autoxidation reactions occur in unsaturated and aldehydic RO_2_, but the model lacks detailed data on RC(O)O_2_ with these substituents. Individual computational rate coefficients collected from a variety of sources^5,16–18^ disagree with the H-SAR predictions mainly within a factor of 5, though with two significant outliers: it overestimates both the aldehydic H-shift rates calculated by da Silva^16^ and Møller *et al.*^18^ by two orders of magnitude (see Section S1 of the ESI†). Unsaturated RO_2_ may also undergo ring closure reactions, which also leads to autoxidation,^19,20^ for which another structure–activity relationship (from now on R-SAR) has been developed by Vereecken *et al.*,^21^ but here the data on RC(O)O_2_ is even more scarce. Computational ring closure rates with both the inner and outer C=C carbons for a single RC(O)O_2_ radical are provided by Vereecken *et al.*,^21^ who note that the former reaction is 20 times faster than the reaction for the corresponding alkyl RO_2_, whereas the latter is only sped up by a factor of 3. We have no way to know how systematic these differences are without further data. Thus, we conclude that the systematic computations initiated by Seal *et al.*^14^ for H-shift reactions of aliphatic RC(O)O_2_ ought to be extended to unsaturated and aldehydic RC(O)O_2_, with similarly systematic calculations performed on ring closure rates for the unsaturated RC(O)O_2_. The predictions from these calculations would then be used to update both SAR models to represent the decisive role of acyl peroxy radicals more accurately.

**Fig. 1:**
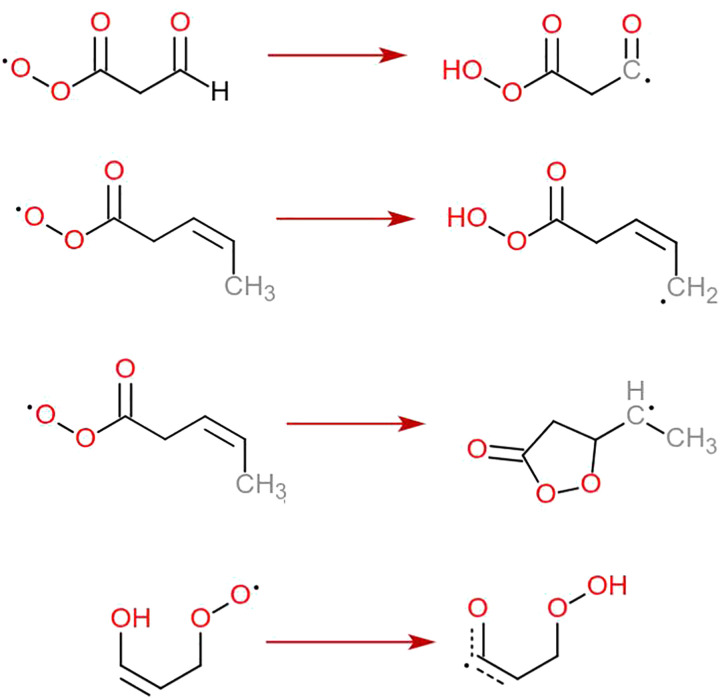
Examples of the four types of autoxidation reactions studied in this work. From top to bottom: Aldehydic H-shifts, allylic H-shifts, and C=C ring closures in RC(O)O_2_, and enolic H-shifts in RO_2_.

In addition to RC(O)O_2_, there is another class of RO_2_ currently not represented in the SAR models but known to autoxidize rapidly: enol-substituted RO_2_. Peeters & Nguyen discovered an exceptionally rapid H-shift for an isoprene-derived non-acyl RO_2_ for the *Z*-isomer but not the *E*-isomer of the enol C=C bond.^22^ The necessity to include this reaction in atmospheric modelling was acknowledged not only by Vereecken & Nozière,^15^ but also by Jenkin *et al.* in their review of the most important RO_2_ reactions to include in automatic mechanism generation.^4^ Despite this, we have very little data on how rapidly other enol-substituted RO_2_ autoxidize. As with the unsaturated and aldehyde-substituted RC(O)O_2_, our GECKO-A-based large exploration of potential RO_2_ + RO_2_ reactions^23^ tentatively indicated that other enol-substituted RO_2_ structures do form downstream from OH addition to organics with multiple C=C bonds (see Section S1 in the ESI†). Thus, we also performed a set of calculations on enol-substituted RO_2_ to extend the H-SAR further. All four reaction types discussed in this work are presented in Fig. 1.

## 2 Methods

In order to extend the aforementioned SAR models, we calculated multi-conformer transition state theory^24,25^ (MCTST) reaction rates in the temperature interval 200–400 K for a total of 78 reactions: 8 H-shifts from aldehyde groups, 14 H-shifts from allylic carbons, 40 ring closures in unsaturated RC(O)O_2_, and 16 H-shifts from enol groups. In our selection of reactions, we have considered all the most impactful functionalities in the existing SAR models, and selected one simple RC(O)O_2_ to represent all other RC(O)O_2_ with the same functionality. No additional calculations were performed to validate our extended SAR models with, but considering the general scarcity of literature data on functionalized RC(O)O_2_, we consider this the most economical usage of computational resources. Our procedure for calculating these MCTST rates largely follows the cost-efficient workflow developed by Møller *et al.*^24^ with minor modifications to further increase the cost-efficiency. The MCTST rates are calculated using the equation:1
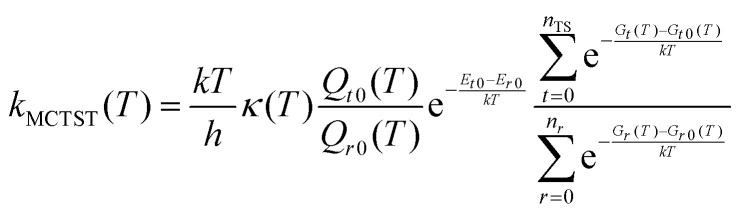
where all the temperature-dependent variables have been explicitly marked for maximum clarity. In the equation, *k* and *h* are the Boltzmann and Planck constants, *T* is the temperature, *κ* is the tunneling coefficient, and *Q* is the thermodynamic partition function, whereas *G* and *E* refer to Gibbs free and zero-point corrected electronic energies, respectively. The *r* and *t* indices refer to conformers of the reactant and transition state, respectively, with *r*0 and *t*0 referring to the global minimum conformers (*i.e.*, conformers with the lowest value of *G*) at *T* = 298 K. *n*_TS_ and *n*_*r*_ are the numbers of unique TS and reactant conformers retained after our conformer filtering workflow described below. All energetics necessary for determining the MCTST rates were calculated using computational chemistry, using the ORCA program,^26–32^ versions 6.0.0 and 6.0.1. In summary, our workflow for determining the energies and partition functions for reactants was the following:

(1) Metadynamics-based conformer search with the CREST software,^33^ using the GFN1-xTB method^34^ and an energy cutoff of 41.84 kJ mol^−1^ for filtering.

(2) Optimization of all conformers found by CREST with B3LYP-D3/ma-def2-SVP^35–38^ followed by uniqueness filtering using the energy and dipole moment cutoffs 1.5 × 10^−5^ Ha and 1.5 × 10^−2^ D. Conformers more than 10 kJ mol^−1^ above the minimum *E* conformer in electronic energy were also filtered out after this step, as they have a negligible contribution to the quotient of Boltzmann sums in eqn (1) at atmospheric temperatures. The choice of duplicate filtering thresholds and conformer energy cutoff are both based on the work of Møller *et al.*^24^

(3) The remaining conformers are re-optimized with the ωB97X-D3/jun-cc-pVTZ level of theory,^39,40^ after which a frequency analysis is performed. Another uniqueness filtering is performed, this time by visually inspecting all conformer pairs with *E* or *μ* values close to the previously mentioned cutoffs.

(4) A DLPNO-CCSD(T)/aug-cc-pVTZ^41–47^ single point energy calculation is performed for the global minimum conformer, with aug-cc-pVQZ auxiliary basis.

(5) For a single representative reaction of each type, the reoptimization done at step 3 and the single-point calculation at step 4 are redone at the ωB97X-D3/aug-cc-pVTZ, and RI-CCSD(T)-F12/cc-pVDZ-F12^48,49^ levels of theory, respectively. For the latter calculation aug-cc-pVDZ was used as auxiliary basis and cc-pVDZ-F12-CABS as complementary auxiliary basis for the F12 calculation. The observed differences in single point energies Δ*E*_F12-DLPNO_ and vibrational zero-point energies ΔZPE_aug-jun_ are used to scale energies calculated using the cheaper DLPNO and jun-cc-pVTZ up or down when calculating MCTST rates. We call these reactions ‘anchor reactions’.

For determining transition state (TS) energies, largely the same steps were followed, with a few modifications:

(1) First, a ‘TS guess geometry’ was built. A constrained optimization with the B3LYP/def2-SVP level of theory was performed with the interatomic distances most relevant for the reaction (as well as the RO_2_ O–O bond length for additional stability) frozen. This was followed by a saddle point search and frequency analysis, also using B3LYP/def2-SVP, after which it was confirmed that the imaginary normal mode corresponded to the intended reaction.

(2) CREST was again used for conformer search with the B3LYP/def2-SVP saddle point used as starting geometry. Constraints were placed on relevant bond lengths, as well as C–C=C–C dihedral angles for molecules with *Z*/*E*-isomerism, as unwanted interconversions were occasionally observed in the CREST-generated ensembles for these radicals.

(3) With the initial conformer ensemble generated, a constrained optimization followed by saddle point search and frequency analysis was performed for all conformers using B3LYP-D3/ma-def2-SVP. Uniqueness filtering was performed similarly as for the reactant conformers.

(4) All unique conformers with *E* or *G* (*T* = 298 K) values within 10 kJ mol^−1^ of the global minimum TS were re-optimized at the ωB97X-D3/jun-cc-pVTZ level of theory, followed by another frequency analysis and another round of uniqueness filtering.

(5) Again, a DLPNO-CCSD(T)/aug-cc-pVTZ single point calculation with aug-cc-pVQZ auxiliary basis was performed for the global minimum conformer.

(6) For anchor reactions, ωB97X-D3/aug-cc-pVTZ reoptimization of the final set of TS conformers and a RI-CCSD(T)-F12/cc-pVDZ-F12 single point energy calculation with aug-cc-pVDZ auxiliary basis and cc-pVDZ-F12-CABS complementary auxiliary basis for the global minimum TS.

For brevity, we will be referring to the RI-CCSD(T)-F12/cc-pVDZ-F12//ωB97X-D3/aug-cc-pVTZ and DLPNO-CCSD(T)/aug-cc-pVTZ//ωB97X-D3/jun-cc-pVTZ levels of quantum theory as F12//aug and DLPNO//jun, respectively. The level-of-theory-based CCSD(T) single point and ZPE corrections for the reactants and TS may be combined into a single ‘TS energy shift’:

2 Δ*E*_ts_ = Δ*E*_ts0,F12-DLPNO_ + ΔZPE_ts0,aug-jun_ − Δ*E*_*r*0,F12-DLPNO_ − ΔZPE_*r*0,aug-jun_

All saddle point searches were initialized with a Hessian calculation for improved geometry convergence. The tunneling coefficient *κ*(*T*) was calculated using the Eckart approach^50,51^ for the reaction coordinate of the lowest *G* (298 K) transition state. As H-shift reaction rates are especially sensitive to tunneling, a few additional calculations were performed to accurately represent the energetics of the reaction coordinate. An intrinsic reaction coordinate^52^ (IRC) calculation was performed, using the B3LYP-D3/ma-def2-SVP level of theory starting from the geometry and Hessian obtained from the saddle point optimization at that level of theory. The reactant and product geometries obtained from the IRC were then reoptimized with ωB97X-D3/jun-cc-pVTZ followed by a DLPNO single point energy calculation (for anchor reactions F12//aug energies were also calculated). These zero-point corrected electronic energies, along with the TS imaginary frequency, were used as parameters for the Eckart *κ*(*T*) calculation.

All thermodynamic partition functions were calculated using the rigid-rotor harmonic oscillator (RRHO) approximation for the vibrational frequencies. Being aware of this model's limitations, we experimented with Grimme's quasi-harmonic oscillator approach, in which the vibrational entropy of each normal mode is calculated with an interpolation function that treats high frequency vibrations as harmonic oscillators and low-frequency vibrations as free rotors.^53^ However, as detailed in Sections S2 and S3 of the ESI,† this approach did not improve our rate calculations’ accuracy. Furthermore, we found that naively applying Grimme's correction only to the vibrational entropy but not the enthalpy (as suggested in the original source) leads to unphysical errors, which at certain frequency ranges could outweight the inaccuracies of the RRHO approximation. Nevertheless, rigorous benchmarking of the accuracy of various approaches to calculating *Q* is beyond the scope of this work.

In order to benchmark our ability to make accurate rate predictions with this computational workflow, we performed test calculations for three RO_2_ H-shift reactions whose rates have already been experimentally constrained. These were the aldehydic H-shift in HOCH_2_C(CH_3_)(CHO)O_2_ measured by Crounse *et al.*,^54^ the allylic H-shift in *Z*-HOCH_2_C(CH_3_)=CHCH_2_O_2_ measured by Teng *et al.*,^55^ and the H-shift from the α-OH carbon in CH_3_CH(OH)CH_2_CH(C_2_H_5_)O_2_ measured by Praske *et al.*^56^ These were selected due to the relative similarity to the RC(O)O_2_ reactions considered in this work. In Section S2 of the ESI† we compare our MCTST calculations to these experimental rates. We found that while reaction rates calculated using the DLPNO//jun energies agree with the experiments within a factor of 4, the rates using the more expensive F12//aug methods reach an agreement within a factor of 2. This observation resulted in workflow described above, in which DLPNO//jun energies were calculated for all reactions, with F12//aug energies calculated for a few anchor reactions. We also find that our usage of B3LYP-D3/ma-def2-SVP rather than B3LYP/6-31+G* (used by Møller *et al.*^24^) improves the accuracy of the low-cost conformer filtering step of the workflow.

### 2.1 Additional notes on conformer sampling

**Fig. 2:**
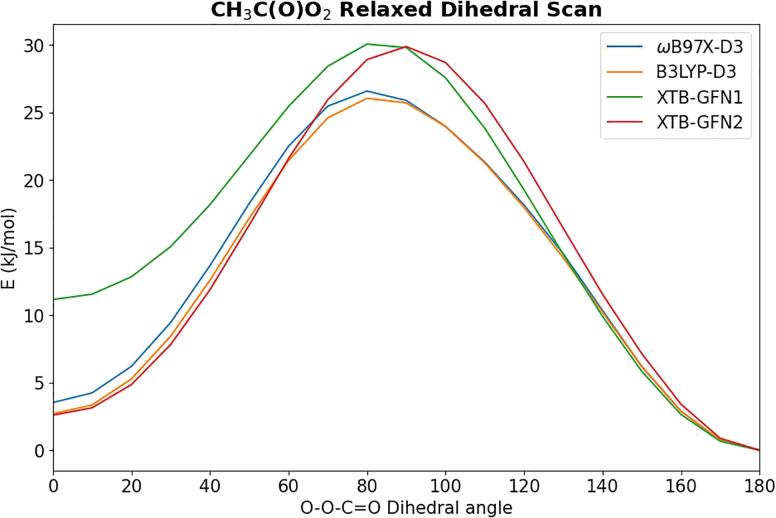
Relaxed surface scan of the acetyl peroxy radical dihedral angle with GFN1-xTB, GFN2-xTB, B3LYP-D3/ma-def2-SVP, and ωB97X-D3/jun-cc-pVTZ.

In previous works from our research group involving conformer sampling,^7,57–59^ we have typically used the molecular mechanics force field in the commercial Spartan software for initial conformer ensemble generation. In this work, we opted for the open source software CREST in order to experiment with sampling methods that do not rely on explicit treatment of covalent bonds. As all the reactions treated in this work are unimolecular isomerizations, they provide a suitable model system where it is relatively easy to tell if crucial bond rotations are missed by the sampling algorithm. As CREST is only compatible with the GFN family of semi-empirical methods, we opted to use the GFN1-xTB method for conformer sampling, as GFN2-xTB has been observed to perform poorly for H-bonded acid–base clusters,^60^ despite reportedly outperforming GFN1-xTB in established benchmark datasets for non-covalent interactions.^61^ We see this as evidence that GFN1-xTB is more robust than GFN2-xTB, and thus more reliable for sampling H-bonding interactions in system well outside the GFN reference molecules, ‘predominantly of closed-shell character and covering common bonding situations.’^34^ In some initial test calculations for RC(O)O_2_ we observed a conspicuous lack of C–O bond rotations in the acyl peroxy functional group, and opted therefore to perform a relaxed surface scan of the O–O–C=O dihedral angle in CH_3_C(O)O_2_ with both GFN methods and DFT methods (Fig. 2) to access the thermal accessibility of these rotations. We found that the torsional barrier was around 25 kJ mol^−1^ according to both B3LYP-D3/ma-def2-SVP and ωB97X-D3/jun-cc-pVTZ, indicating that these rotations should indeed be included in the conformer ensemble. Both XTB methods overestimate the barrier by around 5 kJ mol^−1^, and it appears that this prevents the CREST conformer sampling runs from accessing these rotations when the default energy filter of around 25 kJ mol^−1^ is used. Fig. 2 also shows that GFN1-xTB gets the relative energy difference of the two conformers wrong by 6 kJ mol^−1^, which is roughly in line with the mean average error of 7–16 kJ mol^−1^ for relative conformer energies observed in our benchmarking (see Section S2 in the ESI†). Due to this, we increased CREST's energy cutoff to 41.84 kJ mol^−1^ (‘–ewin 10’ in the input) to ensure that all thermally relevant conformers are found.

A few of the systems studied in this work turned out to be so unstable on the GFN1-xTB PES that the optimization preceding the metadynamics simulation always resulted in an unwanted reaction. In some cases this could be solved by rotating the input geometry into an unreactive conformer, but this occasionally led to crucial reactive conformers (such as the IRC reactant) missing from the final conformer ensemble. The systems for which this was the case were CH_2_=C(CH_3_)CH_2_C(O)O_2_, CH_2_=C(C_2_H_5_)CH_2_C(O)O_2_, CH_2_=C(i-C_3_H_7_)CH_2_C(O)O_2_, and all *Z*-enol-RO_2_. In these cases, we attempted to perform the conformer sampling with the GFN-FF forcefield^62^ as recommended by the CREST output, as it disallows changes to bond topology. However, this approach often led to physical inconsistencies in the reactive energetics, such as the IRC reactant having a lower energy than the lowest-energy reactant conformer found after the DFT re-optimizations were performed on top of the GFN-FF conformer ensemble. We interpreted this as a sign that many of the potential wells on the DFT PES do not exist on the GFN-FF PES, leading to systematic errors when trying to use the latter for conformer sampling. This observation is consistent with a recent performance comparison of low-cost conformer sampling methods,^63^ in which GFN-FF on average failed to find a third of the potential wells on higher-level PES of model radical systems. For the set of problematic radicals listed above, an additional conformer search was performed using ORCA 6.0's new Global Optimizer GOAT,^64^ again using GFN1-xTB. This approach proved much more consistent at generating full conformer ensembles for these radicals, despite utilizing the same level of theory as CREST. This is likely due to differences in the conformer search algorithm: CREST's atomistic metadynamics simulations coupled with automatic filtering of reacted structures likely leads it to miss conformers with especially low reactive barriers on the utilized level of theory. GOAT, on the other hand, freezes all covalent bond lengths and dihedral angles of strong sp^2^ bonds during the conformer search to prevent these unwanted reactions from occurring altogether.^64^ These experiences have led us to believe that the latter approach is more suited to atmospheric organic radicals and other systems with low-barrier reactions, especially if they are not well represented by the benchmark sets used to parametrize the GFN methods.^34^

The conformer ensembles generated with GOAT were optimized using B3LYP-D3/ma-def2-SVP, after which uniqueness filtering was performed to determine which conformers in the GOAT conformer ensemble included structures not present in the CREST/GFN-FF ensemble after B3LYP-D3/ma-def2-SVP optimization. After this, our normal conformer filtering workflow was resumed from step 3 for reactant conformers and step 4 for TS conformers. For clarity, conformers located using GOAT but not CREST are respectively labelled ‘ReacG’ or ‘TSG’ in our ESI.†

## 3 Computational results

### 3.1 Aldehydic H-shifts

Our MCTST results for aldehydic H-shifts are presented in Table 1. The simplest possible RC(O)O_2_ in which an aldehydic H-shift is possible is the 2-formyl acyl peroxy radical, CHOC(O)O_2_, for which G. Silva has previously calculated a H-shift rate using a higher level of quantum theory than ours: G3SX//B3LYP/6-31G(2df,p)^16^ fitting master equation results over a temperature range from 150 K to 400 K, he obtained an Arrhenius expression of 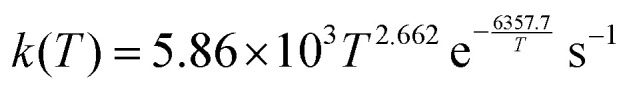. Silva's results are likely more accurate than our MCTST calculation for the same radical in terms of energetics and fall-off effects, but his calculations do not consider multi-conformer effects. Therefore, uniquely for this reaction we derive the SAR expression by applying a *T*-dependent multi-conformer correction to Silva's rate expression:3
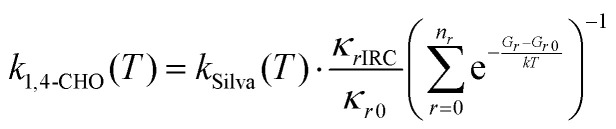
where *κ*_*r*IRC_ is our Eckart tunneling coefficient calculated from the IRC reactant conformer, whereas *κ*_*r*0_ is the tunneling coefficient calculated from the global minimum conformer, which corresponds well to the Silva's CHOC(O)O_2_ geometry.^16^ In this calculation we used the frequency *ω̃* = −1143.56 cm^−1^, determined from B3LYP/6-31G(2df,p) frequency analysis on top of Silva's TS geometry. 
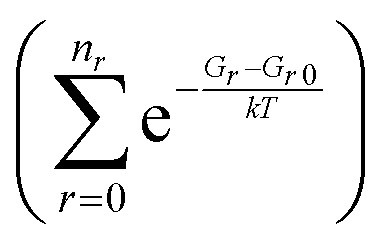
 includes only multi-conformer effects from the reactant side as there is only one TS conformer. Our MCTST correction resulted in a slightly lower rate coefficient, scaling Silva's rates down by a factor between 0.65 at 200 K and 0.42 at 400 K. Slightly different Arrhenius parameters (see Table 8) and a 298 K rate coefficient of 6.20 s^−1^ were obtained as a result.

**Table 1:** Multi-conformer transition state theory reaction rates for aldehydic H-shifts in RC(O)O_2_ and monosubstituted RO_2_ at *T* = 298 K along with the most important parameters for determining the rates. All energies are presented in unit kJ mol^−1^. When calculating *k*_MCTST_ rates for the non-anchor reactions, the Δ*E*_ts_ shift −2.58 kJ mol^−1^ was used for the RC(O)O_2_ reactions and the Δ*E*_ts_ shift −2.22 kJ mol^−1^ for non-RC(O)O_2_ reactions

Radical	Structure	*E*_ts_ − *E*_*r*0_	*G*_ts_ − *G*_*r*0_	*ω̃* (cm^−1^)	*E*_ts_ − *E*_rirc_	*E*_ts_ − *E*_pirc_	*κ*	*k*_MCTST_ (s^−1^)
CHOC(O)O_2_**(Silva)**^16^	1,4-CHO	65.27	70.64	−1143.56^a^				6.20^b^
CHOC(O)O_2_**(F12)**	1,4-CHO	69.91	70.60	−1153.33	67.33	90.92	5.25	7.01
CHOC(O)O_2_**(DLPNO)**	1,4-CHO	77.39	79.97	−1164.78	74.42	97.54	5.59	2.52 × 10^−1^
CHOCH_2_C(O)O_2_**(F12)**	1,5-CHO	69.64	74.97	−1641.45	66.51	74.97	51.08	8.93
CHOCH_2_C(O)O_2_**(DLPNO)**	1,5-CHO	72.21	77.61	−1642.82	70.58	80.08	57.70	3.19
CHOC_2_H_4_C(O)O_2_	1,6-CHO	69.14	76.32	−1530.55	60.35	65.24	22.91	9.12
CHOC_3_H_6_C(O)O_2_	1,7-CHO	66.48	71.80	−1473.13	48.13	60.66	14.89	2.40 × 10^1^
CHOC_4_H_8_C(O)O_2_	1,8-CHO	66.14	74.79	−1516.29	60.17	71.84	22.32	1.76 × 10^1^
CHOC_4_H_8_CH_2_O_2_**(F12)**	1,8-CHO	70.63	80.06	−1981.53	57.34	48.17	143.08	1.01
CHOC_4_H_8_CH_2_O_2_**(DLPNO)**	1,8-CHO	72.86	82.15	−1985.27	59.76	51.96	176.58	4.82 × 10^−1^
CHOC_4_H_8_CH(CH_3_)O_2_	1,8-CHO	75.15	84.16	−2000.03	61.06	49.97	155.78	7.22 × 10^−1^
CHOC_4_H_8_C(CH_3_)_2_O_2_	1,8-CHO	74.85	83.52	−2015.01	60.20	47.97	149.52	6.01 × 10^−1^

a Determined by calculating the B3LYP/6-31G(2df,p) Hessian at Silva's published TS geometry.^16^

b Rate calculated using eqn (3).

For the same reaction, there was a significant discrepancy between our F12//aug and DLPNO//jun results, stemming from a combination of CCSD(T) single points, relative conformer energies and vibrational partition functions. As we already obtained an accurate SAR rate for the 1,4-CHO H-shift from the comparison with Silva's results, we opted to instead use the 1,5-CHO H-shift in CHOCH_2_C(O)O_2_ as an anchor reaction. Here we obtained much better agreement between the F12//aug and DLPNO//jun results. Thus, the Δ*E*_ts_ correction obtained from this reaction was used for the aldehydic H-shifts of span 6, 7 and 8. Since the H-SAR lacked rate expressions for aldehydic H-shifts of span 8, we also calculated reference rates for primary, secondary and tertiary alkyl RO_2_, with the first of these used as an anchor reaction.

When comparing our results to those already in the H-SAR,^15^ we note first that our 1,8-CHO rate for CHOC_4_H_8_CH_2_O_2_ calculated using the F12//aug energetics is approximately half that of Vereecken's recommended 1,7-CHO rate for Prim-RO_2_, based on his MCTST calculation using CBS-QB3//B3LYP/6-31G(d,p) energetics on CHOC_3_H_6_CH_2_O_2_. Notably, this is the only 1,7-CHO rate in the H-SAR reference data, with the recommended rates for *sec*-RO_2_ and *tert*-RO_2_ being based on linear extrapolations of relative rates. This makes it all the more significant that our directly calculated 1,8-CHO rates for *sec*-RO_2_ and *tert*-RO_2_ (0.722 and 0.601 s^−1^ at 298 K, respectively) exceed the H-SAR for the corresponding 1,7-CHO reactions (0.171 and 0.032, respectively), implying that the true reaction rates for the latter may in fact be higher than the current SAR predictions. By comparison, our calculations for CHO-substituted RC(O)O_2_ are slower than the previous H-SAR predictions, and show a different span dependence, peaking at 7 rather than 5 (using the H-SAR's generic RC(O)O_2_ factor of 
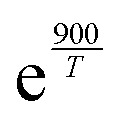
) or 4 (using Seal's span-dependent correction factors^14^). Taken as a whole, the data in Table 1 demonstrate a delicate balance between energetic, entropic, and tunneling contributions to the reaction rate, all of which have a span dependence of their own.

### 3.2 Allylic H-shifts

Our MCTST results for allylic H-shifts are presented in Table 2. The combination of the H-SAR parameters for allylic H-shifts^15^ and the span-dependent RC(O)O_2_ parameters from Seal *et al.*^14^ predict 298 K rates on the order of 10^2^ to 10^3^ s^−1^, with the most extreme rate prediction being 1.16 × 10^6^ s^−1^ for 1,7-CH shifts with endocyclic C=C bonds. Taken at face value, this rate would imply that certain allylic H-shifts in unsaturated RC(O)O_2_ may even outcompete H-shifts from –OOH groups, which are conventionally viewed to outcompete all other RO_2_ reactions.^65^ By comparison, our MCTST rates are much more modest, most fitting within the ‘competitive but not immediate’ range between 10^−3^ and 10^2^ s^−1^. These results seem to imply that the parameters of Seal *et al.* are only applicable to RC(O)O_2_ with flexible carbon backbones.

**Table 2:** Multi-conformer transition state theory reaction rates for H-shifts from allylic carbons in RC(O)O_2_ at *T* = 298 K along with the most important parameters for determining the rates. All energies are presented in unit kJ mol^−1^. When calculating *k*_MCTST_ rates for the non-anchor reactions, the Δ*E*_ts_ shift 1.75 kJ mol^−1^ was used for the gemini-C=C H-shifts, the Δ*E*_ts_ shift 2.02 kJ mol^−1^ for *endo*-C=C H-shifts, and the Δ*E*_ts_ shift 1.30 kJ mol^−1^ for *exo*-C=C H-shifts

Radical	Structure	*E*_ts_ − *E*_*r*0_	*G*_ts_ − *G*_*r*0_	*ω̃* (cm^−1^)	*E*_ts_ − *E*_rirc_	*E*_ts_ − *E*_pirc_	*κ*	*k*_MCTST_ (s^−1^)
CH_2_=C(CH_3_)C(O)O_2_**(F12)**	1,5-CH_3_-*gem*	104.92	109.42	−1987.73	90.75	90.99	1229.60	4.02 × 10^−4^
CH_2_=C(CH_3_)C(O)O_2_**(DLPNO)**	1,5-CH_3_-*gem*	103.17	107.31	−1997.63	89.49	93.77	1375.90	9.13 × 10^−4^
CH_2_=C(C_2_H_5_)C(O)O_2_	1,5-CH_2_-*gem*	89.71	95.59	−1903.95	75.16	91.16	445.30	4.05 × 10^−2^
CH_2_=C(i-C_3_H_7_)C(O)O_2_	1,5-CH-*gem*	77.08	81.03	−1740.96	64.95	91.91	108.23	1.07
CH_2_=C(CH_3_)CH_2_C(O)O_2_	1,6-CH_3_-*gem*	84.49	90.32	−2052.14	79.59	91.69	1566.13	2.03 × 10^−1^
CH_2_=C(C_2_H_5_)CH_2_C(O)O_2_	1,6-CH_2_-*gem*	70.73	75.65	−1944.43	67.88	90.47	445.10	2.26 × 10^1^
CH_2_=C(i-C_3_H_7_)CH_2_C(O)O_2_	1,6-CH-*gem*	62.11	68.37	−1785.52	56.64	85.34	104.64	1.14 × 10^2^
*Z*-CH_3_CH=CHC(O)O_2_**(F12)**	1,6-CH_3_-*endo*	97.33	107.21	−2126.48	82.12	93.13	2791.38	2.80 × 10^−3^
*Z*-CH_3_CH=CHC(O)O_2_**(DLPNO)**	1,6-CH_3_-*endo*	95.31	105.15	−2136.59	80.82	95.24	2968.05	4.91 × 10^−3^
*Z*-C_2_H_5_CH=CHC(O)O_2_	1,6-CH_2_-*endo*	82.30	91.40	−2022.18	68.42	97.52	834.68	2.29 × 10^−1^
*Z*-i-C_3_H_7_CH=CHC(O)O_2_	1,6-CH-*endo*	74.52	84.10	−1874.81	61.21	90.41	219.44	8.45 × 10^−1^
*Z*-CH_3_CH=CHCH_2_C(O)O_2_	1,7-CH_3_-*endo*	80.47	90.15	−2042.67	75.36	96.02	1328.76	2.56 × 10^−1^
*Z*-C_2_H_5_CH=CHCH_2_C(O)O_2_	1,7-CH_2_-*endo*	68.39	76.64	−1911.6	67.25	98.37	378.32	1.67 × 10^1^
*Z*-i-C_3_H_7_CH=CHCH_2_C(O)O_2_	1,7-CH-*endo*	62.10	69.87	−1752.06	63.57	87.64	109.53	1.08 × 10^2^
CH_2_=CHCH_2_C_3_H_6_C(O)O_2_**(F12)**	1,7-CH_2_-*exo*	65.49	79.32	−1811.99	57.26	87.93	116.96	1.17 × 10^1^
CH_2_=CHCH_2_C_3_H_6_C(O)O_2_**(DLPNO)**	1,7-CH_2_-*exo*	64.33	74.36	−1810.71	56.78	91.32	115.78	2.23 × 10^1^
CH_2_=CHCH(CH_3_)C_3_H_6_C(O)O_2_	1,7-CH-*exo*	59.76	70.61	−1682.08	46.40	82.52	42.25	7.41 × 10^1^

**Fig. 3:**
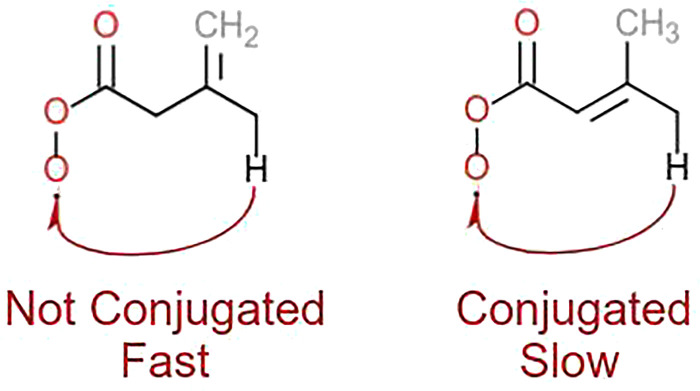
A visual explanation for the observed trends in the allylic 1,6-CH_*n*_ H-shifts.

Another notable detail in our results is the significant difference between the 1,6-CH_*n*_ H-shifts with endocyclic C=C bonds and those with geminal C=C bonds (*i.e.*, with one sp^2^ carbon inside the TS ring and the second outside). The H-SAR treats these two structures as equivalent, but from these results it is evident that the π-orbital conjugation of the C=C bond and the RC(O)O_2_ group plays a role for these systems (see Fig. 3). This finding is partially in line with Vereecken & Nozière's speculation on the impact of conjugated double-bonds rendering allylic H-shifts energetically unfavourable but entropically favourable.^15^ At least for these conjugated RC(O)O_2_ radicals we observe the former but not the latter effect.

Another detail of note regarding allylic H-shifts is that Nozière and Vereecken have recently performed an experimental validation study of both the H-SAR and R-SAR for unsaturated RO_2_, in which the predicted allylic H-shift rates were often found to be too high.^66^ In the article they discuss updating these allylic H-shift parameters in a companion paper, which is unpublished at the time of writing. A proper comparison of our allylic H-shifts trends in RC(O)O_2_ with those in non-acyl RO_2_ may thus have to wait.

### 3.3 Enolic H-shifts

For these reactions we performed an extra set of benchmarking calculations for the three radicals treated by Peeters & Nguyen, in which our F12//aug and DLPNO//jun results are compared against their CBS-QB3//UB3LYP/6-31+G** results.^22^ This comparison is found in Table 3, whereas our MCTST calculations for a set of monosubstituted enol-RO_2_ are presented in Table 4.

**Table 3:** Multi-conformer transition state theory reaction rates computed with our methods for the enol-substituted RO_2_ also studied by Peeters and Nguyen,^22^ along with comparison to the results reported in the cited work

Radical	*E*_ts_ − *E*_*r*0_	*G*_ts_ − *G*_*r*0_	*ω̃* (cm^−1^)	*E*_ts_ − *E*_rirc_	*E*_ts_ − *E*_pirc_	*κ*	*k*_MCTST_ (s^−1^)
*Z*-HOCH=CHCH_2_O_2_**(Peeters)**	48.91		−1614.01	53.09	56.78		
*Z*-HOCH=CHCH_2_O_2_**(F12)**	57.37	60.20	−2270.52	57.37	57.91	810.72	1.33 × 10^5^
*Z*-HOC=C(CH_3_)CH(CH_2_OH)O_2_**(Peeters)**	42.59		−1543.33	33.81	65.27	15.38	2.00 × 10^6^
*Z*-HOC=C(CH_3_)CH(CH_2_OH)O_2_**(F12)**	47.85	49.87	−2326.02	39.99	64.78	346.70	7.57 × 10^6^
*Z*-HOC=C(CH_3_)CH(CH_2_OH)O_2_**(DLPNO)**	53.85	55.87	−2326.02	44.98	71.88	565.75	1.10 × 10^6^
*Z*-HOC=CHC(CH_3_)(CH_2_OH)O_2_**(Peeters)**	51.17		−1632.21	51.17		20.77	6.23 × 10^4^
*Z*-HOC=CHC(CH_3_)(CH_2_OH)O_2_**(F12)**	61.71	64.22	−2328.58	52.14	60.06	859.08	3.07 × 10^4^
*Z*-HOC=CHC(CH_3_)(CH_2_OH)O_2_**(DLPNO)**	66.38	68.74	−2330.25	56.42	66.62	1322.16	7.30 × 10^3^

**Table 4:** Multi-conformer transition state theory reaction rates for H-shifts from enol groups in RO_2_ at *T* = 298 K along with the most important parameters for determining the rates. All energies are presented in unit kJ mol^−1^. The Δ*E*_ts_ shift −3.805 kJ mol^−1^ was used when calculating the *k*_MCTST_ for the non-anchor reactions

Radical	Structure	*E*_ts_ − *E*_*r*0_	*G*_ts_ − *G*_*r*0_	*ω̃* (cm^−1^)	*E*_ts_ − *E*_rirc_	*E*_ts_ − *E*_pirc_	*κ*	*k*_MCTST_ (s^−1^)
*Z*-HOCH=CHCH_2_O_2_**(F12)**	1,6-Prim	57.37	60.20	−2270.52	57.37	57.91	810.72	1.33 × 10^5^
*Z*-HOCH=CHCH_2_O_2_ (DLPNO)	1,6-Prim	61.18	64.09	−2272.21	61.18	64.04	1203.13	4.12 × 10^4^
*Z*-HOCH=CHCH(CH_3_)O_2_	1,6-*sec*	58.46	61.35	−2203.1	58.32	58.45	470.13	2.20 × 10^5^
*Z*-HOCH=CHC(CH_3_)_2_O_2_	1,6-*tert*	57.61	60.50	−2234.65	57.61	61.80	587.20	4.20 × 10^5^
*Z*-HOCH=C(CH_3_)CH_2_O_2_	1,6-Prim (Subst)	53.80	56.47	−2356.43	53.79	71.53	947.02	2.76 × 10^6^
*Z*-HOCH=C(CH_3_)CH(CH_3_)O_2_	1,6-*sec* (Subst)	50.83	53.38	−2273.04	50.83	60.27	455.72	4.21 × 10^6^
*Z*-HOCH=C(CH_3_)C(CH_3_)_2_O_2_	1,6-*tert* (Subst)	47.00	49.64	−2351.62	47.00	69.75	511.18	1.99 × 10^7^
*Z*-HOCH=CHCH_2_CH_2_O_2_	1,7-Prim	61.15	67.75	−2839.02	61.14	86.27	17 152	1.78 × 10^5^
*E*-HOCH=CHCH_2_CH_2_O_2_	1,7-Prim (E)	134.32	141.47	−1569.89	131.97	142.57	103.44	1.48 × 10^−10^
*Z*-HOCH=CHCH_2_CH(CH_3_)O_2_	1,7-*sec*	61.20	66.84	−2842.91	60.69	82.89	15 848	4.12 × 10^5^
*Z*-HOCH=CHCH_2_C(CH_3_)_2_O_2_	1,7-*tert*	64.41	67.49	−2880.57	61.56	82.26	20 206	8.68 × 10^5^
*Z*-HOCH=CHC_2_H_4_CH_2_O_2_	1,8-Prim	60.40	63.77	−2119.7	41.73	69.01	143.94	1.96 × 10^4^
*E*-HOCH=CHC_2_H_4_CH_2_O_2_	1,8-Prim (E)	76.16	86.48	−2032.88	69.58	85.56	580.74	2.45
*Z*-HOCH=CHC_2_H_4_CH(CH_3_)O_2_	1,8-*sec*	49.49	57.91	−2118.65	39.42	67.77	119.38	1.16 × 10^5^
*Z*-HOCH=CHC_2_H_4_C(CH_3_)_2_O_2_	1,8-*tert*	51.40	58.02	−2178.14	38.93	65.22	137.78	2.65 × 10^5^

The most notable difference between our results and those reported by Peeters & Nguyen are that our barrier heights and imaginary frequencies are higher, especially the latter. We suspect the latter difference is due to Peeters's use of B3LYP for frequency analysis. As shown in Table 1 of Peeters and Nguyen,^22^ UB3LYP/6-31+G** on its own underestimates the barrier height for *Z*-HOCH=CHCH_2_O_2_ by about half compared to CBS-QB3//UB3LYP/6-31+G** (26.99 *vs.* 48.91 kJ mol^−1^). The barrier being that much lower on the B3LYP PES implies that it is also less steep, resulting in an underestimation of the imaginary frequency. By comparison, the ωB97X-D3/aug-cc-pVTZ barrier for the same molecule is 52.56 kJ mol^−1^, which is much closer to the values calculated with more accurate levels of theory. Thus we assume that our imaginary frequencies are more accurate. As seen in Table 3, these frequencies lead to significantly higher tunneling corrections, which to some extent compensate for our slightly higher barriers. On the whole however, both ours and Peeters's reaction rates are sufficiently high to outcompete all other irreversible RO_2_ reactions, the fastest being the HO_2_ elimination from RO_2_ with gemini-OH substituents, which has a thermal rate on the order of 10^3^ s^−1^ at 298 K.^67^ Thus, the small observed differences in rates may be seen as insignificant in terms of atmospheric relevance.

Our selection was aimed first to test both the impact of the methyl group in *E* position relative to the enol and the impact of the β-OH group outside the TS cycle present in both of the OH + isoprene-derived RO_2_ studied by Peeters & Nguyen. As expected, the former speeds up the reaction by a factor of 10. The impact of the latter seems less straightforward, as the OH-substituted secondary RO_2_ in Table 3 has an energy barrier 3 kJ mol^−1^ higher than its unsubstituted equivalent, whereas the tertiary RO_2_ has a barrier 9 kJ mol^−1^ above. A second objective was to calculate rates for enol H-shifts at longer spans in order to observe the decay of the reaction rates, and estimate at which spans the mechanism stops mattering. This expected reduction in the H-shift rates is however not present in our results, with the slowest 1,8-*Z*-enol H-shift having a rate of 1.96 × 10^4^ s^−1^ at 298 K and a rate of 2.37 × 10^3^ s^−1^ at 250 K. We conclude that these *Z*-enol H-shift reactions are effectively immediate under all ambient atmospheric conditions, at least in the absence of severe steric hindrance in the molecular structure.

In order to test the impact of this steric hindrance, we also calculated two MCTST rates for the *E* isomers of the Prim-RO_2_ with TS ring spans 7 and 8. Surprisingly, the latter of these turned out to be rapid enough to occur in tropospheric conditions, implying that even *E*-enol H-shifts ought to be considered for RO_2_ where the enol oxygen is attached to the ε-carbon (*e.g.* in HOCH=CHC_2_H_4_CH_2_O_2_) or further. However, we suspect that additional functional groups might provide additional constraints, and thus render these reactions uncompetitive.

### 3.4 Ring closures in β-unsaturated RC(O)O_2_

In the computational reference data for the R-SAR, Vereecken *et al.* calculated 4- and 5-membered ring closure rates for CH_2_=CHCH_2_O_2_, and noted that both rates were far too low to have atmospheric relevance.^21^ Thus, the R-SAR includes no parameters for ring closure rates in β-unsaturated RO_2_. However, in our recent work on the atmospheric oxidation of aromatics we discovered a surprisingly rapid 4-membered ring closure reaction in CH_3_C(O)CH(OH)CH(OOH)C(CH_3_)=CHC(O)O_2_, which forms downstream from OH oxidation of para-xylene.^68^ This implies that 4-membered ring closures may in fact have atmospheric relevance in β-unsaturated RC(O)O_2_, if not in the corresponding non-acyl RO_2_.

In our calculations, we first calculated the 4- and 5-membered ring closure rates for the acryl peroxy radical (CH_2_=CHC(O)O_2_), and used the former as an anchor reaction. This radical has been found to form in isoprene ozonolysis,^69^ and may thus be one of the most abundant RC(O)O_2_ in the troposphere. The 4-membered ring closure was found to be the more competitive reaction, in line with our previous findings.^68^ However, neither of these reactions are rapid enough to compete under atmospheric conditions, implying that ring closures for β-unsaturated systems only become important with the correct substituents. We calculated 4-membered ring closure rates for β-unsaturated RC(O)O_2_ with a similar set of carbon substituents as for the γ and δ-unsaturated RC(O)O_2_ discussed below, in addition to a few other substituted structures that may activate the reaction. Unsurprisingly, we found that the fully substituted (CH_3_)_2_C=C(CH_3_)C(O)O_2_ has the highest reaction rate, along with, somewhat more surprisingly, *Z*-C_2_H_5_CH=C(i-C_3_H_7_)C(O)O_2_ (see Table 5). We suspect that the high amount of branching in the molecular structure of the latter adds steric hindrance to the reactant side that is not equally present in the TS. Generally, it seems that having a carbon substituent on both sides of the C=C bond is enough to bring the room temperature reaction rates above 10^−2^ s^−1^, allowing them to outcompete the lowest known bimolecular RO_2_ decay rates in ambient tropospheric conditions, determined from the rate of RO_2_ + HO_2_ reactions in low NO_*x*_ regimes.^3^

**Table 5:** Multi-conformer transition state theory reaction rates for 4- and 5-membered ring closures in β-unsaturated RC(O)O_2_ at *T* = 298 K along with the most important parameters for determining the rates. All energies are presented in unit kJ mol^−1^. The Δ*E*_ts_ shift 1.11 kJ mol^−1^ was used when calculating the *k*_MCTST_ rates for the non-anchor reactions

Radical	C=C bond, ring size	*E*_ts_ − *E*_*r*0_	*G*_ts_ − *G*_*r*0_	*ω̃* (cm^−1^)	*E*_ts_ − *E*_rirc_	*E*_ts_ − *E*_pirc_	*κ*	*k*_MCTST_ (s^−1^)
CH_2_=CHC(O)O_2_**(F12)**	β-CH=CH_2_, 4	95.88	99.26	−586.65	94.95	60.18	1.44	2.00 × 10^−5^
CH_2_=CHC(O)O_2_**(DLPNO)**	β-CH=CH_2_, 4	94.77	97.87	−588.02	94.28	61.94	1.44	3.33 × 10^−5^
CH_2_=CHC(O)O_2_**(F12)**	β-CH=CH_2_, 5	100.41	99.26	−735.73	85.98	122.29	1.80	2.79 × 10^−6^
CH_2_=CHC(O)O_2_**(DLPNO)**	β-CH=CH_2_, 5	98.58	102.93	−740.09	85.25	122.33	1.82	1.31 × 10^−6^
C_2_H_5_CH=CHC(O)O_2_	β-CH=CH(CH_3_)	91.83	93.34	−471.65	82.23	58.16	1.26	1.31 × 10^−4^
C_3_H_7_C(CH_3_)=CHC(O)O_2_	β-CH=C(CH_3_)_2_	83.22	84.56	−392.45	81.90	54.86	1.17	7.51 × 10^−3^
CH_3_C(O)CH=CHC(O)O_2_	β-CH=CHCO	85.11	88.45	−749.42	85.11	78.48	1.85	1.40 × 10^−3^
i-C_3_H_7_C(=CH_2_)C(O)O_2_	β-C(CH_3_)=CH_2_	81.64	84.58	−508.87	69.99	62.67	1.31	9.53 × 10^−3^
*Z*-C_2_H_5_CH=C(CH_3_)C(O)O_2_	β-C(CH_3_)=CH(CH_3_)	74.88	79.98	−427.5	62.17	59.36	1.21	7.08 × 10^−2^
*Z*-C_2_H_5_CH=C(i-C_3_H_7_)C(O)O_2_	β-C(CH_3_)=CH(CH_3_)	64.06	66.76	−356.73	61.13	59.00	1.14	3.37
(CH_3_)_2_C=C(CH_3_)C(O)O_2_	β-C(CH_3_)=C(CH_3_)_2_, 4	63.03	69.06	−369.01	62.22	59.45	1.15	2.98
(CH_3_)_2_C=C(CH_3_)C(O)O_2_	β-C(CH_3_)=C(CH_3_)_2_, 5	70.90	80.58	−320.90	68.98	139.67	1.11	2.06 × 10^−2^
CH_3_CH=C(OH)C(O)O_2_	β-C(OH)=CH(CH_3_)	84.54	86.81	−311.00	69.85	60.26	1.10	2.66 × 10^−3^
C_2_H_5_CH=C(CH_2_OH)C(O)O_2_	β-CC(OH)=CH(CH_3_)	74.15	79.32	−404.76	62.52	61.38	1.18	9.52 × 10^−2^
C_2_H_5_CH=C(CHO)C(O)O_2_	β-C(CHO)=CH(CH_3_)	82.43	85.50	−404.25	78.26	50.86	1.18	5.47 × 10^−3^
CH_2_=CHCH=CHC(O)O_2_	β-CH=CHCH=CH_2_	88.02	90.10	−748.4	80.83	89.58	1.84	1.18 × 10^−3^

Regarding the fate of the highly strained cyclic peresters formed from these reactions, we suspect that the ring closure is eventually followed by a CO_2_ elimination either before or after O_2_ adds to the radical carbon, leaving behind a carbonyl at the carbon where the ring closure occurred. Unfortunately, we were neither able to confirm nor eliminate these suspicions, as the TS proved highly multi-configurational. Nevertheless, we have documented our attempts in Section S5 of the ESI.†

### 3.5 Ring closures in γ and δ-unsaturated RC(O)O_2_

The R-SAR already covers ring closure reactions for γ, δ, and ε-unsaturated non-acyl RO_2_. We performed calculations of all of the equivalent γ- and δ-unsaturated RC(O)O_2_, except for CH_2_=CHCH_2_C(O)O_2_, the radical already treated by Vereecken *et al.*^21^ These MCTST rates are found in Tables 6 and 7.

**Table 6:** Multi-conformer transition state theory reaction rates for 5- and 6-membered ring closures in γ-unsaturated RC(O)O_2_ at *T* = 298 K along with the most important parameters for determining the rates. All energies are presented in unit kJ mol^−1^. The Δ*E*_ts_ shift −0.41 kJ mol^−1^ was used for 5-membered ring closures and the Δ*E*_ts_ shift 0.44 kJ mol^−1^ for 6-membered ring closures when calculating the *k*_MCTST_ rates for the non-anchor reactions

Radical	C=C bond, ring size	*E*_ts_ − *E*_*r*0_	*G*_ts_ − *G*_*r*0_	*ω̃* (cm^−1^)	*E*_ts_ − *E*_rirc_	*E*_ts_ − *E*_pirc_	*κ*	*k*_MCTST_ (s^−1^)
CH_2_=CHCH_2_C(O)O_2_^a^	γ-CH=CH_2_, 5	54.63						1.33 × 10^2^
CH_2_=CHCH_2_C(O)O_2_^a^	γ-CH=CH_2_, 6	72.62						1.08 × 10^−1^
CH_2_=C(CH_3_)CH_2_C(O)O_2_**(F12)**	γ-C(CH_3_)=CH_2_, 5	43.90	48.98	−479.92	39.95	67.27	1.27	6.43 × 10^3^
CH_2_=C(CH_3_)CH_2_C(O)O_2_**(DLPNO)**	γ-C(CH_3_)=CH_2_, 5	44.31	49.13	−480.59	41.05	71.81	1.27	5.07 × 10^3^
CH_2_=C(CH_3_)CH_2_C(O)O_2_**(F12)**	γ-C(CH_3_)=CH_2_, 6	69.10	72.43	−468.98	65.15	87.82	1.26	5.31 × 10^−1^
CH_2_=C(CH_3_)CH_2_C(O)O_2_**(DLPNO)**	γ-C(CH_3_)=CH_2_, 6	68.66	72.23	−470.55	65.61	90.16	1.26	4.95 × 10^−1^
*Z*-CH(CH_3_)=CHCH_2_C(O)O_2_	γ-CH=CH(CH_3_), 5	37.98	45.33	−446.55	36.54	64.73	1.23	7.51 × 10^4^
*Z*-CH(CH_3_)=CHCH_2_C(O)O_2_	γ-CH=CH(CH_3_), 6	66.83	74.80	−468.94	65.39	97.28	1.26	1.98 × 10^−1^
*E*-CH(CH_3_)=CHCH_2_C(O)O_2_	γ-CH=CH(CH_3_), 6	65.24	70.87	−412.64	63.02	93.13	1.19	2.08
*Z*-CH(CH_3_)=C(CH_3_)CH_2_C(O)O_2_	γ-C(CH_3_)=CH(CH_3_), 5	30.82	37.21	−398.15	26.85	66.52	1.18	1.01 × 10^6^
*Z*-CH(CH_3_)=C(CH_3_)CH_2_C(O)O_2_	γ-C(CH_3_)=CH(CH_3_), 6	59.67	65.58	−451.06	55.71	92.90	1.23	8.35
*E*-CH(CH_3_)=C(CH_3_)CH_2_C(O)O_2_	γ-C(CH_3_)=CH(CH_3_), 6	58.66	62.59	−350.75	54.55	92.93	1.13	2.42 × 10^1^
C(CH_3_)_2_=CHCH_2_C(O)O_2_	γ-CH=C(CH_3_)_2_, 5	31.32	40.14	−383.60	32.79	62.25	1.16	2.99 × 10^5^
C(CH_3_)_2_=CHCH_2_C(O)O_2_	γ-CH=C(CH_3_)_2_, 6	55.65	66.56	−406.87	56.95	93.89	1.19	9.09
C(CH_3_)_2_=C(CH_3_)CH_2_C(O)O_2_	γ-C(CH_3_)=C(CH_3_)_2_, 5	26.78	34.31	−324.89	23.31	68.93	1.11	2.79 × 10^6^
C(CH_3_)_2_=C(CH_3_)CH_2_C(O)O_2_	γ-C(CH_3_)=C(CH_3_)_2_, 6	46.97	56.55	−342.14	43.53	96.66	1.13	2.98 × 10^2^

a From Vereecken *et al.*^21^

**Table 7:** Multi-conformer transition state theory reaction rates for 6- and 7-membered ring closures in δ-unsaturated RC(O)O_2_ at *T* = 298 K along with the most important parameters for determining the rates. All energies are presented in unit kJ mol^−1^. The Δ*E*_ts_ shift 1.30 kJ mol^−1^ was used for 6-membered ring closures and the Δ*E*_ts_ shift 1.52 kJ mol^−1^ for 7-membered ring closures when calculating the *k*_MCTST_ rates for the non-anchor reactions

Radical	C=C bond, ring size	*E*_ts_ − *E*_*r*0_	*G*_ts_ − *G*_*r*0_	*ω̃* (cm^−1^)	*E*_ts_ − *E*_rirc_	*E*_ts_ − *E*_pirc_	*κ*	*k*_MCTST_ (s^−1^)
CH_2_=CHC_2_H_4_C(O)O_2_**(F12)**	δ-CH=CH_2_, 6	47.82	55.29	−552.11	41.70	59.35	1.38	4.99 × 10^2^
CH_2_=CHC_2_H_4_C(O)O_2_**(DLPNO)**	δ-CH=CH_2_, 6	46.52	53.98	−552.26	40.94	61.85	1.38	8.99 × 10^2^
CH_2_=CHC_2_H_4_C(O)O_2_**(F12)**	δ-CH=CH_2_, 7	59.81	67.89	−522.12	53.69	71.34	1.33	3.11
CH_2_=CHC_2_H_4_C(O)O_2_**(DLPNO)**	δ-CH=CH_2_, 7	58.28	66.35	−522.88	52.51	73.20	1.33	6.13
CH_2_=C(CH_3_)C_2_H_4_C(O)O_2_	δ-C(CH_3_)=CH_2_, 6	40.74	49.06	−493.49	35.75	61.90	1.29	2.93 × 10^3^
CH_2_=C(CH_3_)C_2_H_4_C(O)O_2_	δ-C(CH_3_)=CH_2_, 7	52.16	59.59	−482.05	47.44	70.23	1.27	4.22 × 10^1^
*Z*-CH(CH_3_)=CHC_2_H_4_C(O)O_2_	δ-CH=CH(CH_3_), 6	38.42	46.09	−478.70	34.16	59.24	1.27	1.43 × 10^4^
*Z*-CH(CH_3_)=CHC_2_H_4_C(O)O_2_	δ-CH=CH(CH_3_), 7	50.19	61.63	−497.33	46.20	77.97	1.29	2.75 × 10^1^
*E*-CH(CH_3_)=CHC_2_H_4_C(O)O_2_	δ-CH=CH(CH_3_), 7	49.53	57.36	−468.61	45.61	71.54	1.26	1.66 × 10^2^
*Z*-CH(CH_3_)=C(CH_3_)C_2_H_4_C(O)O_2_	δ-C(CH_3_)=CH(CH_3_), 6	29.16	36.59	−432.19	27.72	59.47	1.21	4.72 × 10^5^
*Z*-CH(CH_3_)=C(CH_3_)C_2_H_4_C(O)O_2_	δ-C(CH_3_)=CH(CH_3_), 7	41.22	50.23	−452.65	39.66	69.92	1.24	2.23 × 10^3^
*E*-CH(CH_3_)=C(CH_3_)C_2_H_4_C(O)O_2_	δ-C(CH_3_)=CH(CH_3_), 7	41.90	51.74	−404.85	39.85	64.19	1.19	2.17 × 10^3^
C(CH_3_)_2_=CHC_2_H_4_C(O)O_2_	δ-CH=C(CH_3_)_2_, 6	33.49	39.75	−430.54	29.83	55.87	1.21	1.42 × 10^5^
C(CH_3_)_2_=CHC_2_H_4_C(O)O_2_	δ-CH=C(CH_3_)_2_, 7	43.42	53.98	−452.96	39.77	75.81	1.24	3.94 × 10^2^
C(CH_3_)_2_=C(CH_3_)C_2_H_4_C(O)O_2_	δ-C(CH_3_)=C(CH_3_)_2_, 6	22.29	35.49	−348.90	20.21	57.34	1.13	1.71 × 10^6^
C(CH_3_)_2_=C(CH_3_)C_2_H_4_C(O)O_2_	δ-C(CH_3_)=C(CH_3_)_2_, 7	33.08	49.00	−374.14	31.18	62.13	1.16	8.75 × 10^3^

In short, our main conclusion is that unsaturated RC(O)O_2_ indeed systematically prefer forming a ring with the inner sp^2^-carbon, regardless of the substitution around the C=C bond. Also notably, the 5-membered ring closure reactions in the more substituted γ-unsaturated RC(O)O_2_ are sped up by two or three orders of magnitude relative to their respective non-acyl RO_2_. The 6-membered ring closure reactions in δ-unsaturated RC(O)O_2_ are even more rapid relative to their respective non-acyl RO_2_ reactions, usually reaching within an order of magnitude of the corresponding γ-RC(O)O_2_ reactions. Furthermore, the 7-membered ring closure rates in δ-unsaturated RC(O)O_2_ are also significantly sped up relative to their respective non-acyl RO_2_ reactions, while still being essentially uncompetitive compared to the 6-membered ring closures. In short, we observe a similar shift in ring size trends as with the allylic and aldehydic H-shifts, with the peak of the ‘rate as a function of ring size’ curve being shifted to larger ring sizes for RC(O)O_2_ relative to other RO_2_. This is likely a consequence of the C=O group adding additional strain to the smaller rings.

One especially notable detail about these ring closure reactions is that the fastest rates reach the same order of magnitude as the irreversible hydroperoxide H-shift reactions. Unlike the case with the allylic H-shift mentioned above, these rates are directly computed rather than SAR-predicted, giving more credence to the suggestion that these reactions could outcompete the H-shift. To investigate this, we calculated 5-membered ring closure and –OOH H-shift rates for two hypothetical RC(O)O_2_ containing both a γ-C=C bond and a hydroperoxide on either side of the C=C bond. The full results for this are presented in Section S4 of the ESI,† but in short our results suggest that the presence of the C=C bond slows down the H-shifts from the OOH groups. Based on these results, we conclude that ring closures in γ and δ-unsaturated RC(O)O_2_ presumably always outcompete the –OOH H-shift.

## 4 Extension of structure–activity relationships

Our MCTST results for aldehydic and allylic H-shifts as well as the ring closures were used to derive the *T*-dependent SAR parameters, using the same modified Arrhenius expression as in the original H-SAR and R-SAR articles^15,21^ (eqn (4)). The results are found in Tables 8 and 9. In addition, we estimate rate coefficients and *T*-dependent SAR parameters for ε-unsaturated RC(O)O_2_ using the relative rate coefficients of δ- and ε-unsaturated RO_2_ from the R-SAR along with our rates for δ-unsaturated RC(O)O_2_ (eqn (5)).4
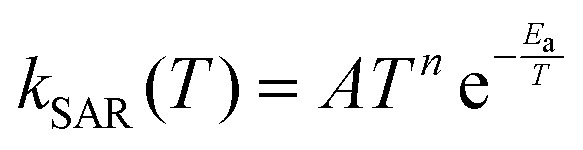
5
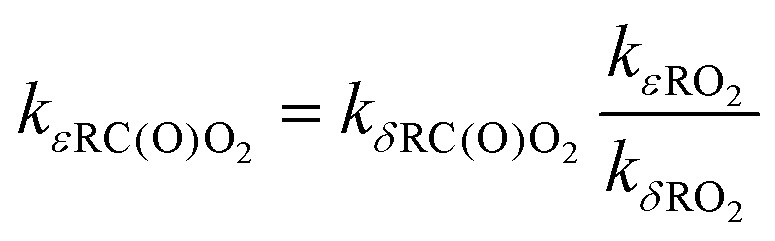


**Table 8:** Temperature-dependent Arrhenius parameters for the H-shift SAR, to be used in the form 
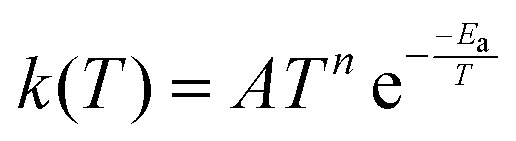

RO_2_	Span & H	*A* (s^−1^)	*n*	*E*_a_ (K)	RO_2_	Span & H	*A* (s^−1^)	*n*	*E*_a_ (K)
RC(O)O_2_	1,4-CHO	2.16 × 10^6^	1.60	6517	RCH_2_O_2_	1,8-CHO	1.92 × 10^−35^	14.26	381
RC(O)O_2_	1,5-CHO	6.67 × 10^−60^	23.03	−2160	RCH(R)O_2_	1,8-CHO	1.80 × 10^−33^	13.63	765
RC(O)O_2_	1,6-CHO	2.12 × 10^−45^	18.24	−340	RC(R)_2_O_2_	1,8-CHO	1.82 × 10^−31^	2.98	1095
RC(O)O_2_	1,7-CHO	2.87 × 10^−30^	13.25	1277	RC(O)O_2_	1,8-CHO	4.21 × 10^−47^	18.82	−720
RC(O)O_2_	1,5-CH_3_-*gem*	1.11 × 10^−77^	28.53	−2047	RC(O)O_2_	1,6-CH_3_-*endo*	3.17 × 10^−65^	23.92	−1896
RC(O)O_2_	1,5-CH_2_-*gem*	7.68 × 10^−71^	26.56	−2074	RC(O)O_2_	1,6-CH_2_-*endo*	1.60 × 10^−58^	21.94	−1819
RC(O)O_2_	1,5-CH-*gem*	1.41 × 10^−63^	24.31	−1867	RC(O)O_2_	1,6-CH-*endo*	2.76 × 10^−56^	21.49	−1587
RC(O)O_2_	1,6-CH_3_-*gem*	1.26 × 10^−69^	25.38	−3805	RC(O)O_2_	1,7-CH_3_-*endo*	1.20 × 10^−64^	23.64	−3332
RC(O)O_2_	1,6-CH_2_-*gem*	9.75 × 10^−61^	22.82	−3360	RC(O)O_2_	1,7-CH_2_-*endo*	2.61 × 10^−65^	24.44	−3669
RC(O)O_2_	1,6-CH-*gem*	5.93 × 10^−51^	19.79	−2280	RC(O)O_2_	1,7-CH-*endo*	8.97 × 10^−64^	24.29	−3421
RC(O)O_2_	1,7-CH_2_-*exo*	1.11 × 10^−49^	19.11	−1876	RC(O)O_2_	1,7-CH-*exo*	4.49 × 10^−38^	15.61	−402

**Table 9:** Temperature-dependent Arrhenius parameters for unsaturated RC(O)O_2_ for the ring closure SAR, to be used in the form 
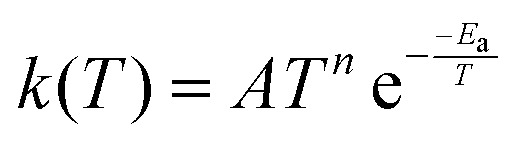

C=C bond, ring size	*A* (s^−1^)	*n*	*E*_a_ (K)	C=C bond, ring size	*A* (s^−1^)	*n*	*E*_a_ (K)
β-CH=CH_2_, 4	8.80 × 10^7^	1.32	10 921	β-CH=CH_2_, 5	4.75 × 10^3^	2.61	10 770
β-C(CH_3_)=CH_2_, 4	2.84 × 10^9^	0.98	9543	β-CH=CH(CH_3_), 4	2.40 × 10^10^	0.67	10 917
β-CH=C(CH_3_)_2_, 4	1.01 × 10^11^	0.61	10 041	β-C(CH_3_)=CH(CH_3_), 4	6.57 × 10^6^	1.93	8742
β-C(CH_3_)=C(CH_3_)_2_, 4	9.40 × 10^10^	0.25	7621	β-C(CH_3_)=C(CH_3_)_2_, 5	1.24 × 10^11^	−0.10	8598
γ-CH=CH_2_, 5^a^	8.05 × 10^8^	0.65	5757	γ-CH=CH_2_, 6^a^	3.05 × 10^11^	−0.16	8271
γ-C(CH_3_)=CH_2_, 5	7.68 × 10^10^	−0.03	4808	γ-C(CH_3_)=CH_2_, 6	5.89 × 10^11^	−0.20	7920
γ-CH=CH(CH_3_), 5	8.97 × 10^10^	0.02	4205	*Z*-γ-CH=CH(CH_3_), 6	4.02 × 10^10^	−0.08	7626
				*E*-γ-CH=CH(CH_3_), 6	2.54 × 10^10^	0.40	7599
γ-C(CH_3_)=CH(CH_3_), 5	3.53 × 10^10^	0.12	3315	*Z*-γ-C(CH_3_)=CH(CH_3_), 6	1.23 × 10^10^	0.30	6805
				*E*-γ-C(CH_3_)=CH(CH_3_), 6	1.68 × 10^13^	−0.70	6941
γ-CH=C(CH_3_)_2_, 5	1.62 × 10^10^	0.07	3362	γ-CH=C(CH_3_)_2_, 6	2.88 × 10^9^	0.25	6259
γ-C(CH_3_)=C(CH_3_)_2_, 5	1.04 × 10^11^	−0.14	2894	γ-C(CH_3_)=C(CH_3_)_2_, 6	6.74 × 10^9^	0.14	5288
δ-CH=CH_2_, 6	1.16 × 10^9^	0.47	5169	δ-CH=CH_2_, 7	3.94 × 10^8^	0.61	6604
δ-C(CH_3_)=CH_2_, 6	2.32 × 10^9^	0.25	4470	δ-C(CH_3_)=CH_2_, 7	6.39 × 10^9^	0.20	5952
δ-CH=CH(CH_3_), 6	1.77 × 10^10^	0.13	4394	*Z*-δ-CH=CH(CH_3_), 7	1.09 × 10^8^	0.64	5615
				*E*-δ-CH=CH(CH_3_), 7	1.14 × 10^9^	0.56	5634
δ-C(CH_3_)=CH(CH_3_), 6	2.52 × 10^10^	0.05	3322	*Z*-δ-C(CH_3_)=CH(CH_3_), 7	3.60 × 10^9^	0.33	4920
				*E*-δ-C(CH_3_)=CH(CH_3_), 7	3.89 × 10^9^	0.32	4841
δ-CH=C(CH_3_)_2_, 6	1.20 × 10^6^	1.65	3432	δ-CH=C(CH_3_)_2_, 7	5.17 × 10^8^	0.34	4781
δ-C(CH_3_)=C(CH_3_)_2_, 6	4.47 × 10^9^	0.03	2397	δ-C(CH_3_)=C(CH_3_)_2_, 7	1.65 × 10^8^	0.39	3603
ε-CH=CH_2_, 7	2.51 × 10^10^	−0.07	6324	ε-CH=CH_2_, 8	2.05 × 10^4^	1.75	5358
ε-C(CH_3_)=CH_2_, 7	1.07 × 10^11^	−0.39	5571	ε-C(CH_3_)=CH_2_, 8	3.79 × 10^5^	1.30	4719
ε-CH=CH(CH_3_), 7	9.35 × 10^9^	0.14	5330	*Z*-ε-CH=CH(CH_3_), 8	1.33 × 10^3^	2.00	4346
				*E*-ε-CH=CH(CH_3_), 8	1.66 × 10^3^	2.20	4029
ε-C(CH_3_)=CH(CH_3_), 7	5.76 × 10^9^	0.17	4071	*Z*-ε-C(CH_3_)=CH(CH_3_), 8	1.27 × 10^4^	1.92	3714
				*E*-ε-C(CH_3_)=CH(CH_3_), 8	4.28 × 10^5^	1.42	3418
ε-CH=C(CH_3_)_2_, 7	4.51 × 10^5^	1.72	4503	ε-CH=C(CH_3_)_2_, 8	1.89 × 10^3^	1.90	3528
ε-C(CH_3_)=C(CH_3_)_2_, 7	2.84 × 10^8^	0.30	3020	ε-C(CH_3_)=C(CH_3_)_2_, 8	2.68 × 10^3^	1.63	1955

a From Vereecken *et al.*^21^

Similarly, we note that Carter *et al.*^70^ have used the reference data of Vereecken & Nozière along with the computed reaction rates of Møller *et al.*^18^ to extrapolate the reaction rates predicted by the H-SAR up to arbitrarily high H-shift spans. Something similar may be done to estimate rates for RC(O)O_2_ H-shifts with higher spans than those calculated here, but our view is that such an estimation ought to also include not only our results, but also the computational results of Seal *et al.*^14^ and the forthcoming extended H-SAR by Vereecken & Nozière^66^ as reference data. Thus, we will perform this extrapolation in a future paper, in which we introduce both extended SAR models into the automatic mechanism generator GECKO-A.^71^

We opted not to derive SAR parameters for the *Z*-enol H-shifts, as our recommendation for these reactions is to simply assume that they are always instantaneous, as all of our computed reaction rates are well above those of any competing RO_2_ reactions. As noted in Section 3.3, *E*-enol H-shifts may also prove important for larger molecules with long and flexible carbon backbones between the RO_2_ and enol groups. We have not derived SAR parameters for these either, as we are unsure to which extent our linear, monofunctional model radicals represent true atmospheric radicals, as these are likely to have multiple substituents. We may revisit the subject when such data is available.

In terms of atmospheric significance of our results, we expect the aldehydic H-shift parameters to be an especially crucial refinement of the H-SAR, as aldehydes occur commonly in highly oxidized RO_2_ structures.^23^ Additionally, as we already noted in Section 3.1, our computational results are in some ways more complete than the reference data that informed the original SAR, which only included *sec*- and *tert*-RO_2_ with short H-shift spans. We find that a re-parametrization of the aldehydic H-shifts may also be in order for non-acyl RO_2_. For unsaturated RC(O)O_2_ with at least one carbon between the C=O and C=C bond, our findings suggest that ring closure reactions, especially with the inner sp^2^ carbon, are overwhelmingly the major fate in atmospheric conditions. For β-unsubstituted RC(O)O_2_, on the other hand, allylic H-shifts and 4-membered ring closure reactions may both be competitive depending on the molecular structure. Nevertheless, the allylic H-shifts are also a valuable addition to the H-SAR for completion purposes, despite the fact that they seem to be outcompeted by the ring closures in most cases.

Due to the scarcity of literature rates of unimolecular reactions in functionalized acyl peroxy radicals (let alone experimentally constrained literature rates, which do not exist to our knowledge), we do not have much data to validate our SAR extensions with. This is especially true for ring closure reactions, for which the sole unsaturated RC(O)O_2_ system studied by Vereecken *et al.*^21^ is already incorporated into our R-SAR extension. For H-shifts, however, we may use the computational literature rates in Table S1 (ESI†) to tentatively estimate the applicability of the extended H-SAR for arbitrary RC(O)O_2_ with the correct substituents. For the sole aldehydic H-shift rate calculated by Møller *et al.*,^18^ which the original H-SAR overestimates by a factor of 124 at 298 K, our extended H-SAR predicts a rate of 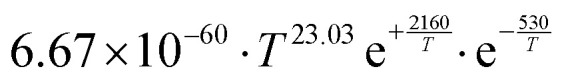, where the second exponential function is the β-OH factor from Vereecken and Nozière.^15^ This results in a rate of 1.51 s^−1^ at 298 K, which overestimates Møller's directly calculated rate by a factor of 5.8. While not ideal, this disagreement is largely in line with the others in Table S1 (ESI†), for which the maximum is 7.69 and the geometric mean is 2.59. We thus assume that the extended SAR models predict rates for arbitrary RC(O)O_2_ within an order of magnitude at atmospheric temperatures.

## 5 Conclusions

In this work, we have extended the structure–activity relationships for unimolecular reactions of RO_2_ developed by Vereecken and coworkers to several classes of rapid reactions that were previously only rudimentarily included in the SAR: H-shifts in CHO-substituted RC(O)O_2_, allylic H-shifts and ring closures in unsaturated RC(O)O_2_, and H-shifts in enol-substituted RO_2_. These groups of radicals are among the shortest-lived of all RO_2_ in the atmosphere, and we therefore find it crucial that their fates are accurately represented by the SAR. While deriving this SAR extension, we have also gained new mechanistic insight on the reactivity trends of substituted acyl peroxy radicals.

In the process of calculating the reactions kinetics for all these unimolecular reactions, we have also further improved the cost-effective conformer sampling workflow of Møller *et al.*^24^ Based on our experiences, we recommend using ORCA's new global optimizer code GOAT for generation of an initial conformer ensemble, and performing low-level filtering optimizations with B3LYP-D3 rather than B3LYP. Furthermore, ωB97X-D(3)/aug-cc-pVTZ optimizations may be swapped out for ωB97X-D(3)/jun-cc-pVTZ optimizations, and CCSD(T)-F12 single-points for DLPNO-CCSD(T) single points, but both of these changes come with a minor decrease in accuracy.

## Author contributions

LF performed the quantum chemistry calculations, MCTST rate calculations, derived the SAR parameters, and drafted the manuscript. AS performed the quantum chemistry calculations on the 4-membered ring closures. SI, MR & TK provided consultation and useful discussions. The manuscript was reviewed and revised by all authors.

## Conflicts of interest

There are no conflicts to declare.

## Supplementary Material

CP-027-D5CP01175B-s001

## Data Availability

Multi-conformer transition state theory reaction rates from 200 K to 400 K, ORCA output files for all published results, as well as our bash-wrapped python script for calculating the former from the latter, are freely available in a F.A.I.R Zenodo repository: https://zenodo.org/records/15089952.
