# Supplementary material for: Rapid unimolecular reactions of acyl peroxy radicals: extending the structure–activity relationships
Source: Phys Chem Chem Phys. 2025 Jun 3;27(23):12198–210. doi: 10.1039/d5cp01175b (PMC12132090; doi:10.1039/d5cp01175b)
Supplement: CP-027-D5CP01175B-s001 [file CP-027-D5CP01175B-s001.pdf]

Supplementary Information (SI) for Physical Chemistry Chemical Physics.
This journal is © the Owner Societies 2025

Electronic Supplementary Information for Rapid unimolecular reactions of acyl peroxy radicals: extending the structure-activity relationships

Lauri Franzon*, Anni Savolainen, Siddharth Iyer, Matti Rissanen, Theo Kurtén*

Contents

S1 Comparison of SAR Predictions with literature rates S1

S2 Benchmarking of multi-conformer workflow S3

S3 Theoretical treatment of the vibrational partition function S5

S4 Competition between hydroperoxide H-shifts and ring closures S8

S5 In search of a TS for CO_2 elimination S11

S1 Comparison of SAR Predictions with literature rates

In Vereecken & Nozière's original formulation of the RO_2 H-shift SAR (H-SAR),[1] the suggested treatment for $RC(O)O_2$ reactions was to simply scale the H-shift rates of prim-RO_2 up by a factor of $e^{\frac{900\ K}{T}}$. The extension of the H-SAR by Seal *et al.*[2] proved that this correction factor is accurate for H-shifts with spans 1,5 and 1,6, but that H-shifts with shorter or longer spans must be corrected by a higher factor. Comparing the H-SAR predictions to the computational H-shift rates for multifunctional $RC(O)O_2$ with these spans reported by da Silva[3], Rissanen *et al.*[4], Barua *et al.*[5] and Møller *et al.*[6], we concluded that applying these updated span parameters together with other H-SAR substituent factors seems to work reasonably well for α-OOH, β-C=O as well as α-OH and β-OH H-shift positions, but not for aldehydic H-shifts. This data is presented in Table S1.

Source	Label in source	H	Subst.	Span	MCTST (s^{-1})	H-SAR (s^{-1})	Disagreement
[4]	$k_f(3B)$	CH	α-OOH	1,7	3.80	3.90	1.03
[4]	H-shift	CH	α-OOH	1,4	$1.10 \cdot 10^{-4}$	$7.00 \cdot 10^{-5}$	1.57
[4]	$k_f(5a)$	CH_2	exo-oxo	1,7	$4.50 \cdot 10^{-2}$	$1.72 \cdot 10^{-1}$	3.82
[4]	$k_f(3a)$	CHO	β-OOH	1,8	$5.00 \cdot 10^{-1}$	-	-
[6]	k_{1B}	CH_3	endo-oxo	1,6	$5.50 \cdot 10^{-3}$	$7.16 \cdot 10^{-4}$	7.69
[6]	k_{2B}	CH_3	β-OH	1,5	$4.40 \cdot 10^{-4}$	$8.40 \cdot 10^{-5}$	5.24
[6]	k_{2C}	CH_2OH	α&β-OH	1,5	$3.00 \cdot 10^{-2}$	$2.75 \cdot 10^{-2}$	1.09
[6]	k_{3B}	CH_3	β-OH	1,5	$3.50 \cdot 10^{-4}$	$8.40 \cdot 10^{-5}$	4.17
[6]	k_{3C}	CHO	β-OH	1,5	$2.60 \cdot 10^{-1}$	32.3	124.10
[3]	TS2	CHO		1,4	6.20^a	$7.69 \cdot 10^2$	123.98
[5]	C4 Channel	CH	α-OOH	1,6	2.08	1.17	1.78

Table S1: A collection of computationally determined H-shifts rates in multifunctional $RC(O)O_2$ from the literature. All presented rates are at temperature 298 K. The SAR rate for reaction $k_f(3a)$ is missing, as Vereecken and Nozière[1] only report SAR parameters for aldehydic H-shift spans up to 1,7. The disagreement is defined as the quotient of MCTST and H-SAR rates.
[a] See Section 3.1 in the main article how this rate was determined.

As we see from Table S1, most of the H-SAR predictions land within an order of magnitude of the computational literature rates, which is most notable for the reactions with span 7 or 8, for which the updated $RC(O)O_2$ span parameters from Seal *et al.*[2] are used. However, there is a large disagreement between the H-SAR and MCTST rates for the aldehydic H-shift. We see two possible explanations for this: Either the rate-inhibiting $e^{\frac{-530\ K}{T}}$ factor for β-OH substituents[1] underestimates the inhibition for aldehydic H-shifts, or the H-SAR values generally overestimates the rate of aldehydic H-shifts for $RC(O)O_2$. The latter interpretation is supported by the surprisingly low MCTST rate for $k_f(3a)$, as the available H-SAR-predicted values for spans 1,4 to 1,7 are all on the order of 10^2 s^{-1}.[1] Furthermore, $RC(O)O_2$ with C=C bonds are completely missing from the reference data.

To further justify the importance of extending the SAR models, we will review the datasets published in our previous work[7] to determine how commonly these C=C and CHO-substituted $RC(O)O_2$, as well as the enol-substituted RO_2, form in atmospheric oxidation (Table S2). Here it seems that $RC(O)O_2$ with C=C and CHO substituents are not uncommon in monoterpene oxidation, at least compared to other $RC(O)O_2$. The numbers may be small compared to the total number of RO_2 produced, but not insignificantly small. As discussed in the main text, the fates of these multifunctional $RC(O)O_2$ has an disproportionately large impact on the organic accretion product formation, which further justified our interest in performing explicit calculations on them.

[1]Admittedly the β-OOH substituent, which lacks a parameter in the H-SAR, may also slow down the rate similarly to a β-OH substituent. In light of our computational rate of $1.76 \cdot 10^1$ s^{-1} in Table 1 of the main text, this may indeed be the case.

Dataset	Terp		DTA			
Generation	1st	2nd	1st	2nd	3rd	4th
Total RO_2	302	3801	82	878	2185	3544
Enol-RO_2	18	102	6	23	41	55
$RC(O)O_2$	26	402	11	114	385	776
with C=C	9	91	3	20	37	99
with CHO	6	139	2	38	139	252

Table S2: The numbers of RO_2, enol-substituted RO_2, $RC(O)O_2$, and $RC(O)O_2$ with the specific substituents formed in the GECKO-A mechanisms in our previous work.[7] The Terp dataset has isoprene, α-pinene, β-pinene, Limonene, Sabinene, Δ-Carene, β-Ocimene, and Myrcene as RO_2 precursors, whereas the DTA dataset had n-Decane, Toluene and α-pinene. The C=C and CHO substituted $RC(O)O_2$ typically formed in the oxidation of compounds with multiple C=C bonds, such as Limonene, β-ocimene and Toluene.

S2 Benchmarking of multi-conformer workflow

The average accuracy of the relative conformer energies for each low-level optimization method relative to ωB97X-D3/aug-cc-pVTZ is presented for the aldehydic H-shift in $HOCH_2C(CH_3)(CHO)O_2$ measured by Crounse *et al.*[8] the allylic H-shift in Z-$HOCH_2C(CH_3)$=$CHCH_2O_2$ measured by Teng *et al.*[9], and the H-shift from the α-OH carbon in $CH_3CH(OH)CH_2CH(C_2H_5)O_2$ measured by Praske *et al.*[10] in Table S3. Time durations are also presented for the optimization and frequency analysis ('Property time' in the ORCA output, which in practice ought to correspond to the calculation time of the Hessian). As the time duration of computational chemistry calculations is dependent on the allocated hardware resources, we will also report that all of the benchmarking calculations were performed on four cores. We do not have fully comparable durations for the GFN1-xTB optimizations, as these were done in CREST. However, for comparison the 'multi-level optimization wall time', combined for all conformers and rotamers, reported by the program were 00:02:41, 00:04:51, and 00:12:15 for the three reactions. These calculations were also performed on four cores.

From these results we conclude that B3LYP-D3/ma-def2-SVP is indeed an improvement[2] compared to the previously used B3LYP/6+31-G*, that large energy cutoffs in CREST are necessary to reproduce accurate conformer ensembles, and that the partially augmented jun-cc-pVTZ basis set is indeed perfectly able to reproduce the relative conformer energies of aug-cc-pVTZ in the higher-level ωB97X-D3 optimization.

The accuracy of MCTST rates compared to experiments are presented in Table S4, varying both the level of quantum theory used in calculating the energetics and the statistical method used to calculate the vibrational partition function. The latter are all calculated using the F12/aug energies and frequencies. 'TightOpt' and 'VeryTightSCF' refer to convergence criteria in ORCA 6.0.0. The former decreases the geometry root mean square displacement gradient threshold from 10^{-4} to $3 \cdot 10^{-5}$ Bohrs among other adjustments, and the latter decreases the SCF energy tolerance from 10^{-8} to 10^{-9} Hartree, among other adjustments. 'Naive QHO' and 'Corr. QHO' refer to two different approaches to the quasi-harmonic approximation, fully explained in Section S3. The 25 or 100 in parenthesis refer to the interpolation parameter $\tilde{\tau}$.

[2]Except for Crounse's RO_2 ($HOCH_2C(CH_3)(CHO)O_2$), for which B3LYP/ma-def2-SVP performed marginally better than B3LYP-D3/ma-def2-SVP. We assume this is due to the D3 dispersion correction producing artefacts due not being developed for small molecules. Nevertheless, as both errors were below 0.6 kJ/mol, we do not consider this an argument against using the D3 correction for conformer filtering.

System	Level of Theory	MAD ($\frac{kJ}{mol}$)	Cycles	Opt time/cycle	Prop. time
Crounse conf=14, uconf=8	ωB97X-D3/aug-cc-pVTZ	-	2.4	0:19:57	0:13:32
	ωB97X-D3/jun-cc-pVTZ	0.026 (0.043)	6.6	0:05:45	0:09:44
	B3LYP-D3/ma-def2-SVP	0.545 (1.271)	13.3	0:00:58	-
	B3LYP/ma-def2-SVP	0.392 (1.113)	14.1	0:00:46	-
	B3LYP/6+31-G*	0.769 (1.561)	14	0:00:56	-
	GFN1-xTB	7.824 (19.220)	-	-	-
Teng conf=25, uconf=12	ωB97X-D3/aug-cc-pVTZ	-	1.8	0:22:29	0:16:10
	ωB97X-D3/jun-cc-pVTZ	0.046 (0.132)	7.3	0:07:43	0:12:19
	B3LYP-D3/ma-def2-SVP	0.513 (2.100)	18.2	0:01:09	-
	B3LYP/ma-def2-SVP	1.621 (9.769)	18.3	0:00:51	-
	B3LYP/6+31-G*	1.724 (10.103)	18.4	0:00:44	-
	GFN1-xTB	16.009 (32.190)	-	-	-
Praske conf=261, uconf=74	ωB97X-D3/aug-cc-pVTZ	-	1.8	1:03:54	0:36:10
	ωB97X-D3/jun-cc-pVTZ	0.053 (0.400)	7.2	0:11:00	0:15:52
	B3LYP-D3/ma-def2-SVP	1.076 (4.964)	12.4	0:01:35	-
	B3LYP/ma-def2-SVP	2.350 (7.275)	12.7	0:01:04	-
	B3LYP/6+31-G*	2.892 (8.804)	13.2	0:01:00	-
	GFN1-xTB	6.828 (16.241)	-	-	-

Table S3: Averaged conformer mean absolute deviation (MAD) from the ωB97X-D3/aug-cc-pVTZ relative energy (maximum error in parenthesis) and average optimization time per optimization cycle for every level of theory utilized for the benchmark systems. The time format is hh:mm:ss. conf: Number of conformers found by CREST. uconf: Number of unique conformers.

Rate/Method (s^{-1})	Crounse (296 K)[8]	Teng (297 K)[9]	Praske (296 K)[10]	Praske (318 K)[10]
Exp	0.5 ± 0.3	0.36 ± 0.14	0.048 ± 0.024	0.31 ± 0.12
DLPNO//jun	0.151	0.549	0.0324	0.166
TightOpt + VeryTightSCF	0.149	0.546	0.0322	0.165
DLPNO//aug	0.129	0.387	0.0307	0.158
F12//jun	0.543	0.529	0.0488	0.247
F12//aug	0.472	0.376	0.0466	0.238
HO	0.472	0.376	0.0466	0.238
Naive QHO(100)	0.407	0.467	0.1090	0.577
Naive QHO(25)	0.491	0.312	0.0675	0.349
Corr. QHO(100)	0.563	0.349	0.0718	0.378
Corr. QHO(25)	0.480	0.313	0.0595	0.308

Table S4: Comparison of computational MCTST rates for benchmarking systems with variation of the basis set used for the final ωB97X-D3 geometry optimizations (jun-cc-pVTZ vs aug-cc-pVTZ), approach to single-point CCSD(T) calculations (DLPNO-CCSD(T)/aug-cc-pVTZ vs CCSD(T)-F12/RI/cc-pVDZ-F12 with their respective auxiliary basis sets), convergence thresholds and the calculation methods for the vibrational partition function (See Section S3).

As shown in Table S4, the F12//aug energetics perform systematically better compared to the DLPNO//jun energetics when it comes to agreement with experimental references. Furthermore, for Crounse's and Praske's reactions it is evidently the F12 single-point that brings the computational MCTST rate closer to the experimental value, whereas for Teng's reaction it is the use of aug-cc-pVTZ in the final conformer optimization. As the latter contrasts with the excellent agreement in relative conformer energies between ωB97X-D3/jun-cc-pVTZ and ωB97X-D3/aug-cc-pVTZ, we took a closer look at the source of the 1.42 factor disagreement between the DLPNO//jun and DLPNO//aug reaction rates at the reference temperature. The

multi-conformer components of the reaction rates $\left(\frac{\sum_{t=0}^{n_{TS}} \mathrm{e}^{-\frac{G_t(T)-G_{t0}(T)}{kT}}}{\sum_{r=0}^{n_r} \mathrm{e}^{-\frac{G_r(T)-G_{r0}(T)}{kT}}}\right)$ only disagreed by a factor of 1.027, further confirming that the change in basis set only had a minor impact on the relative conformer energies. On the other hand, the lowest-conformer partition function ($\frac{Q_{t0}(T)}{Q_{r0}(T)}$) and exponent ($\mathrm{e}^{-\frac{E_{t0}-E_{r0}}{kT}}$) components disagreed by factors of 1.21 and 1.11, respectively. Furthermore, the latter difference is due to a 0.267 kJ/mol disagreement in the DLPNO//aug and DLPNO//jun energy barriers, from which 0.225 kJ/mol comes from the difference in zero point energies. These differences, combined with a minimal difference between the jun- and aug-optimized geometries [3] imply that the additional diffuse functions in aug-cc-pVTZ compared to jun-cc-pVTZ might in some cases be necessary for accurate Hessian calculations, if not conformer filtering.

Compared to the impact of the choice in computational method and basis set, adjustment of the convergence criteria in ORCA had no observable impact on the reaction rates. Furthermore, the treatment of the vibrational partition function only seems to have a minor impact at approximately room temperature. The biggest differences are observed for Praske's reaction, which is to be expected, as the RO_2 in question has both the largest amount of vibrational degrees of freedom (60 as opposed to 39 or 45) and the largest amount of rotable bonds (7 as opposed to 5). However, even here all MCTST rates expect for those using Naive QHO(100) for the vibrational partition function fit within the experimental uncertainty. From this we concluded that the assumption that one can expect a certain amount of error cancellation when using the harmonic oscillator model for room temperature MCTST calculations to be largely correct. On the other hand, it is certainly a larger source of error at the higher end of our temperature range (400 K), as is our usage of a 10 kJ/mol energy cutoff in conformer filtering. This is well beyond the temperature range of the tropospheric conditions where our reactions would be relevant, however, so we find this potential error source acceptable within the scope of this work.

In summary, what these benchmarking calculations have taught us is that a high energy cutoff in CREST is necessary for representative conformer ensembles, that B3LYP-D3/ma-def2-SVP is a good method for pre-optimization of said CREST conformer ensembles, and that ωB97X-D3/jun-cc-pVTZ can perfectly reproduce the relative conformer energies of ωB97X-D3/aug-cc-pVTZ, if not always the Hessians. We have also learned that MCTST rates calculated using the DLPNO//jun energetics reach within a factor of 4 of the experimental references, but that the F12//aug are systematically better, reaching an accuracy within a factor of 2 for the benchmark reactions. This informed the decision described in the main text to calculate the F12//aug energetics for 'anchor reactions', and use the differences in barrier energies to scale energy barriers calculated using DLPNO//jun up or down.

S3 Theoretical treatment of the vibrational partition function

The harmonic oscillator model (HO) is known to overestimate the entropies of low-frequency vibrations such as bond rotations. Grimme suggested fixing this overestimation with a continuous interpolation function returning the HO entropy for higher vibrational frequencies and the one-dimensional rigid rotor entropy for low frequencies,[11] often referred to as the quasi-harmonic oscillator approach (QHO). This approach for correcting HO partition functions has proven quite popular in recent years, due to not requiring additional quantum chemical calculations beyond the Hessian. However, as noted in the main text, we discovered a physical inconsistency in the derivation of the model while experimenting with it for our MCTST calculations, which we will present here.

[3] root mean square distance calculated from the internal geometries in the ORCA output was $5.4 \cdot 10^{-3}$ Å for the global minimum reactant and $3.6 \cdot 10^{-3}$ Å for the global minimum transition state

First of all, the entropy may be defined as follows in statistical mechanics:[12]

$$S(T) = k \ln Q(T) + \frac{E_T(T)}{T} \tag{1}$$

where k is the Boltzmann constant, Q is the thermodynamical partition function, and $E_T(T)$ is the average thermal energy in the canonical ensemble. This component of the entropy is sometimes presented as the T derivative of $\ln Q$. However, we find that such a presentation overlooks a crucial detail when one computes the Gibbs free energy G (assuming the ideal gas law $pV = NkT$ applies):

$$G = H - TS = E_0 + E_T + pV - kT \ln Q - E_T = E_0 + kT(N - \ln Q) \tag{2}$$

where $N = 1$ applies for unimolecular systems such as the ones treated in this work. As seen in the equation, the thermal energy (E_T) cancels out due to being included in both H and S, leaving the zero-point corrected electronic energy (E_0) and the partition function Q as the two relevant quantities for calculating ΔG for unimolecular processes. This results in errors if one calculates H and S using two different approaches, as is implicitly done in the Grimme model, in which the correction is only applied to the vibrational entropy. This correction is based on the previously mentioned harmonic wavenumber ($\tilde{\omega}$) dependent interpolation function $w(\tilde{\omega})$:

$$S_{QHO} = w(\tilde{\omega}) S_{HO} + [1 - w(\tilde{\omega})] S_{r1D} \qquad w(\tilde{\omega}) = \frac{1}{1 + \left(\frac{\tilde{\tau}}{\tilde{\omega}}\right)^4} = \frac{\tilde{\omega}^4}{\tilde{\tau}^4 + \tilde{\omega}^4} \tag{3}$$

where $\tilde{\tau}$ is an interpolation parameter, for which Grimme's original article suggests using a value of 100 cm^{-1},[11] whereas the CREST documentation suggests using a value of 25 cm^{-1}[13] in reference to the work on conformational entropy by Pracht and Grimme[14]. We however fail to find any such recommendations from the specified source.[4] While the HO entropy is conventionally defined as $S_{HO} = -k \ln\left(1 - \mathrm{e}^{-\frac{hc\tilde{\omega}}{kT}}\right) + \frac{hc\tilde{\omega}}{\mathrm{e}^{\frac{hc\tilde{\omega}}{kT}} - 1}$, where h is the Planck constant and c is the speed of light, the 1D rigid rotor entropy S_{r1D} is calculated using an 'effective moment of inertia' μ' computed using the vibrational frequency and the average molecular moment of inertia I_{av}:

$$S_{r1D} = k \ln Q_{r1D} + \frac{k}{2} \qquad \ln Q_{r1D} = \ln\left[\sqrt{\frac{8\pi^3 \mu' kT}{h^2}}\right] \; \mu' = \frac{\mu I_{av}}{\mu + I_{av}} \qquad \mu = \frac{h}{8\pi^2 \omega} \tag{4}$$

where $\frac{k}{2}$ is $\frac{E_T(T)}{T}$ for a 1D rigid rotor. While Equation 4 was expressed using the same notation as Grimme[11], we used a slightly different expression in our own calculations to avoid unit conversions between SI units and wavenumber units as well as floating point errors related to using the former. If both the vibrational frequency $\tilde{\omega}$ and the rotational constants $\tilde{A}$, $\tilde{B}$ and $\tilde{C}$ are expressed in wavenumber units (as is the case in ORCA output), the expressions for I_{av}, μ', and Q^2_{r1D} simplify to:

$$I_{av} = \frac{h}{8\pi^2 c} \times \frac{1}{3}\left(\frac{1}{\tilde{A}} + \frac{1}{\tilde{B}} + \frac{1}{\tilde{C}}\right) \equiv \frac{h}{24\pi^2 c \tilde{\mu}_R} \implies \mu' = \frac{h}{8\pi^2 c \left(3\tilde{\mu}_R + \tilde{\omega}\right)} \tag{5}$$

$$\implies Q^2_{r1D} = \frac{8\pi^3 \mu' kT}{h^2} = \frac{kT}{hc} \frac{\pi}{\left(3\tilde{\mu}_R + \tilde{\omega}\right)} \tag{6}$$

where $\tilde{\mu}_R$ is a parameter we call the 'reduced rotational constant' for its similarity to the reduced mass, $\tilde{\mu}_R = \frac{\tilde{A}\tilde{B}\tilde{C}}{\tilde{A}\tilde{B} + \tilde{A}\tilde{C} + \tilde{B}\tilde{C}}$. We used this expression for calculating the QHO partition functions and entropies in Table S4. Now that we have expressions for both Q_{HO} and Q_{r1D},

[4]Thermochemistry calculations in ORCA use Grimme's QHO method with $\tilde{\tau} = 100$ cm^{-1} by default.

we may apply Grimme's interpolation function to only the $k \ln Q$ component of the vibrational entropy, in accordance with the definition of G in Equation 2. We call this approach **Corrected QHO**. When accounting for all vibrational degrees of freedom, the end result is:

$$\ln Q_{QHO}(\tilde{\tau}) = \sum_{v=1}^{3N-6} -\frac{\tilde{\omega}_v^4}{\tilde{\tau}^4 + \tilde{\omega}_v^4} \ln\left(1 - \mathrm{e}^{-\frac{hc\tilde{\omega}_v}{kT}}\right) + \frac{\tilde{\tau}^4}{\tilde{\tau}^4 + \tilde{\omega}_v^4} \frac{1}{2} \ln\left(\frac{kT}{hc} \frac{\pi}{(3\tilde{\mu}_R + \tilde{\omega}_v)}\right) \tag{7}$$

where we have simplified the expression further by opening up the $w(\tilde{\omega})$ functions. If we instead apply the interpolation to S like suggested in the Grimme model, we get a positive error in G from the thermal energy expression in H being calculated using HO and the thermal energy expression in S being calculated using QHO. The magnitude of this error is:

$$E_{T,HO} - E_{T,QHO} = \sum_{v=1}^{3N-6} E_{T,v} - E_{T,v} w(\tilde{\omega}) - [1 - w(\tilde{\omega})] E_{T,r} = \sum_{v=1}^{3N-6} [1 - w(\tilde{\omega})] (E_{T,v} - E_{T,r})$$
$$= \sum_{v=1}^{3N-6} \frac{\tilde{\tau}^4}{\tilde{\tau}^4 + \tilde{\omega}_v^4} \left[\frac{hc\tilde{\omega}_v}{\mathrm{e}^{\frac{hc\tilde{\omega}_v}{kT}} - 1} - \frac{kT}{2}\right] \neq 0$$

where $E_{T,v}$ and $E_{T,r}$ are the HO and r1D contributions of individual vibrational modes to the thermal energy. As we see, this error is the highest for low-frequency vibrations with a high 1rD character in the interpolation, especially if a relatively high value is chosen for $\tilde{\tau}$ (See Figure S1). We call this approach **Naive QHO**. The difference between HO, Naive QHO, and Corrected QHO per vibrational mode to G as a function of $\tilde{\omega}$ is shown in Figure S1. The rotational constants for the global minimum reactant of CH_2=CHC(O)O_2 were used, resulting in $\tilde{\mu}_R = 0.034841$ cm^{-1}.

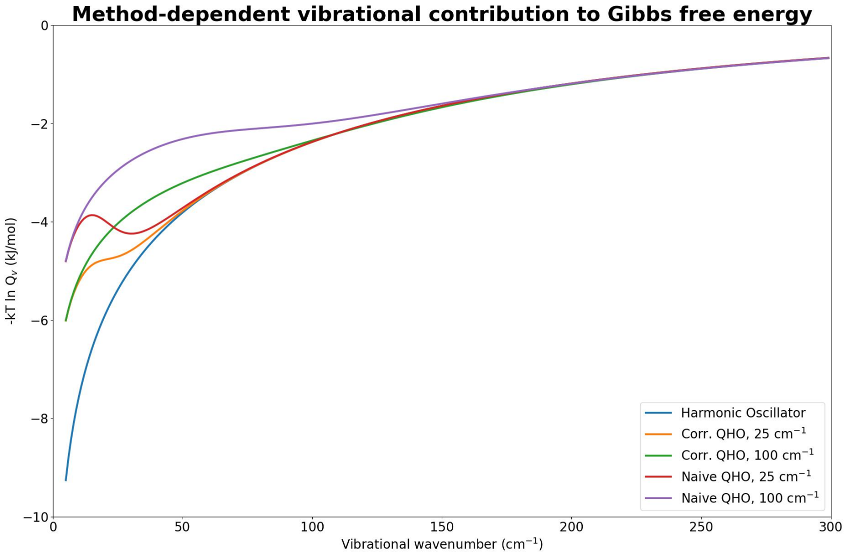

Figure S1: The impact of the choice of vibrational partition function on the vibrational contribution to G at 298 K. As we see, the thermal energy cancellation error starts to be significant around $\tilde{\omega} < 2\tilde{\tau}$, and gets progressively worse for lower frequencies. The kink upward observed around $\tilde{\omega} \approx \frac{1}{2}\tilde{\tau}$ is also an effect of the cancellation error, but its extent also depends on the value of $\tilde{\mu}_R$. Notably though the low-$\tilde{\omega}$ limits of Naive & Corrected QHO agree better with each other than with the HO approximation.

As discussed in Section S2, neither the naive or corrected version of the QHO model improved our MCTST rates compared to the HO model. In fact, using Naive QHO with the interpolation parameter $\tilde{\tau} = 100$ cm^{-1} often made the results worse. Using 25 cm^{-1} instead as recommended

by the CREST manual brings the results more in line with HO, but we find ourselves unable to take this recommendation at face value, since this cited source provided no clues on whether the authors were aware of the thermal energy cancellation error or not. If they were not, we find it likely that such a low parameter value was only found optimal due to low values of $\tilde{\tau}$ minimizing the cancellation error, and as a result rendering the QHO partition functions practically equivalent to HO. Nevertheless, as an additional cautionary note, we would like to report that we found significant differences in how the different approaches for vibrational partition functions order conformers by G, which may impact the final reaction rates in our cost-efficient MCTST workflow, as the wrong conformer may be chosen for CCSD(T) single point calculations. An example of this is shown in Figure S2, in which Naive QHO(100) predicted a different global minimum TS conformer for Crounse's reaction compared to the HO approximation and both QHO(25) approaches. We note that while the thermal energy cancellation error plays into this (the TS conformers all have three vibrational wavenumbers below 200 cm^{-1} but none below 80 cm^{-1}), we see the the same result when using Corrected QHO(100) at room temperature, with the lowest-G conformer according to HO only overtaking the lowest-E_0 conformer at 314 K. This observation underlines the need to benchmark the impact of $\tilde{\tau}$ on the accuracy of the QHO approximation in any form, as the theoretical treatment of the vibrational partition function has an impact on the relative G values of conformers.

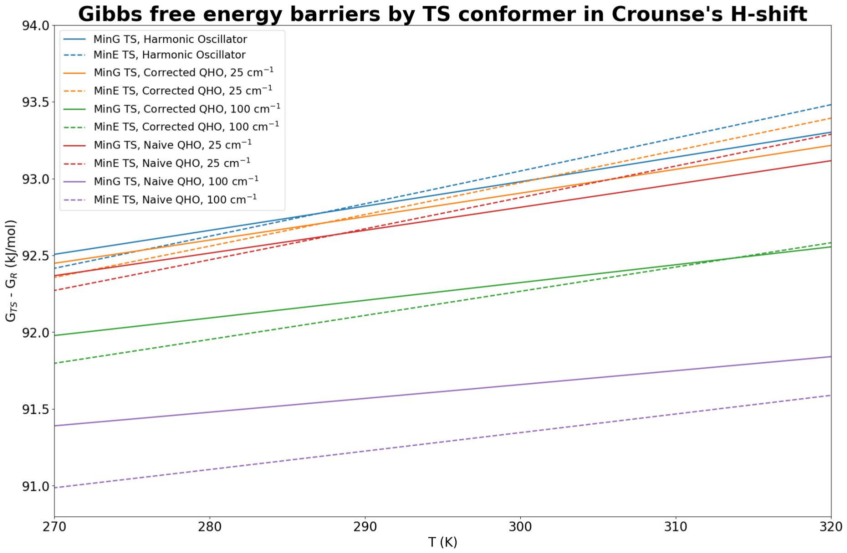

Figure S2: Impact of the theoretical treatment of vibrational corrections to G on the lowest G TS conformer as a function of temperature. Crounse's reaction was chosen to demonstrate this effect as it was one of the reactions for which the minimum E_0 and G(298 K) saddle points differed.

S4 Competition between hydroperoxide H-shifts and ring closures

As mentioned in the main text, the exceptionally rapid ring closure reactions of γ and δ-unsaturated $RC(O)O_2$ raised a suspicion that these reactions may be able to outcompete H-shifts from hydroperoxide groups. We investigated this by performing a few calculations on the smallest possible $RC(O)O_2$ structures containing both a C=C bond in the γ position and an OOH substituent, $CH_3CH{=}CHCH(OOH)C(O)O_2$ and $CH_2(OOH)CH{=}CHCH_2C(O)O_2$. We stress that these specific radicals may not form in the atmosphere, and that they are simply chosen as model structures to test if the hydroperoxide H-shift ought to be seen as instantaneous also for unsaturated $RC(O)O_2$. Nevertheless, in our previous work on the oxidation of para-xylene we predicted the formation of a β-unsaturated $RC(O)O_2$ with a OOH substituent,[15] implying that the formation of $RC(O)O_2$ with both functional groups does indeed occur. For these radicals, only the ring closure with the inner C=C carbon was considered, as this was shown to always be

the faster reaction in our main results.

The results of these calculations are shown in Table S5, and the transition states of each reaction are visualized in Figure S3. As we see, the ring closures are indeed able to outcompete the H-shifts. However, the reason for this is rather surprising: Our H-shift reaction rates are orders of magnitude slower than those reported by Knap and Jørgensen [16]. More specifically, our IRC barrier for the 1,6 H-shift is 3-5 kJ/mol higher than any of their IRC barriers for 1,6 H-shifts, but our reaction rate is lower due to the 13 kJ/mol difference between the IRC reactant and the global minimum reactant. By comparison, our IRC and global minimum reactant energies are only 3 kJ/mol apart for both Z- and E-$CH_2(OOH)CH{=}CHCH_2C(O)O_2$, but here the IRC barriers are respectively 7 and 26 kJ/mol higher than the 1,9 H-shift barrier calculated for $CH_2(OOH)C_3H_6C(O)O_2$ by Knap and Jørgensen. Furthermore, we note that the TS barrier for the H-shift in Z-$CH_2(OOH)CH{=}CHCH_2C(O)O_2$ appears to be thicker than then others, and certainly thicker than Knap and Jørgensen's TS barriers, almost all of which had imaginary wavenumbers around 2500-2600 cm^{-1}. We assume that all of these physical parameters are due to the steric hindrance the C=C bond imposes on molecular structure.

In summary, if $RC(O)O_2$ with both OOH substituents and C=C bonds in γ position or further form in atmospheric conditions, then the ring closure reactions are overwhelmingly the dominant unimolecular reaction. The same may be true for β-unsaturated $RC(O)O_2$ as well, as seen in our work on para-xylene.[15]

Radical	Reaction	$E_{ts} - E_{r0}$	$G_{ts} - G_{r0}$	$\tilde{\omega}$ (cm^{-1})	$E_{ts} - E_{rirc}$	$E_{ts} - E_{pirc}$	κ	k_{MCTST} (s^{-1})
Z-β-OOH	γ-Ring	45.48	47.11	-456.12	38.58	65.99	1.24	$5.68 \cdot 10^4$
Z-β-OOH	1,6-Shift	84.56	86.55	-2571.59	71.26	98.94	27023	$9.10 \cdot 10^1$
Z-ϵ-OOH	γ-Ring	38.26	43.47	-463.37	35.91	69.55	1.25	$1.91 \cdot 10^5$
Z-ϵ-OOH	1,9-Shift	60.77	68.62	-1986.97	57.67	84.89	309	$7.35 \cdot 10^2$
E-ϵ-OOH	γ-Ring	41.12	48.20	-471.31	37.29	65.29	1.26	$1.63 \cdot 10^4$
E-ϵ-OOH	1,9-Shift	79.88	90.05	-2611.87	76.33	101.87	58080	$1.22 \cdot 10^1$

Table S5: Competition between OOH H-shifts and 5-membered ring closure in γ-unsaturated $RC(O)O_2$. Energies are expressed in kJ/mol.
β-OOH: $CH_3CH{=}CHCH(OOH)C(O)O_2$ ϵ-OOH:$CH_2(OOH)CH{=}CHCH_2C(O)O_2$

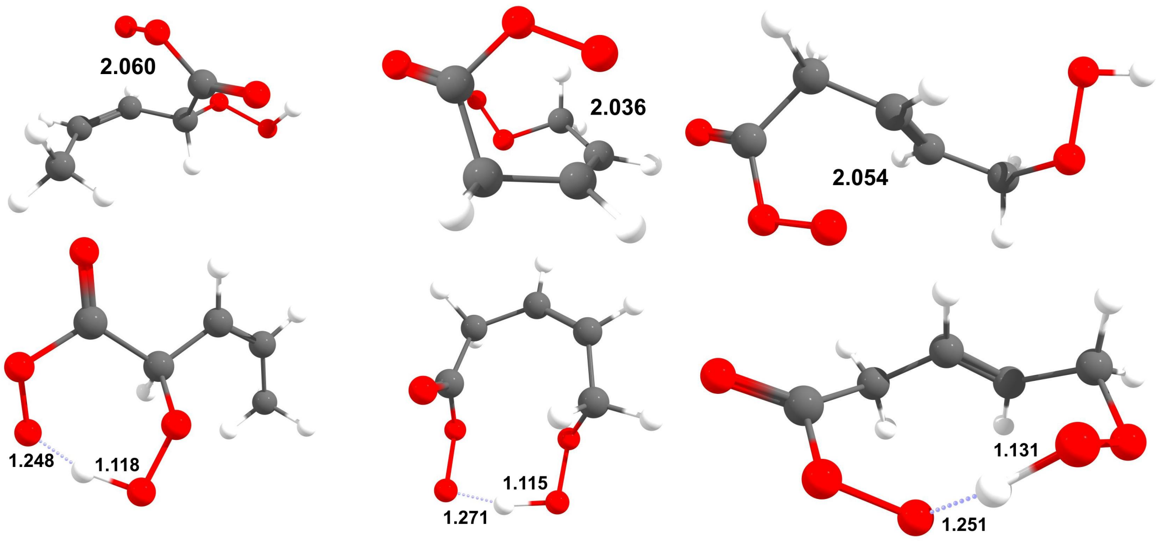

Figure S3: Visualisations of the transition states for ring closure (above) and H-scramble (below) for $CH_3CH{=}CHCH(OOH)C(O)O_2$, (left), Z-$CH_2(OOH)CH{=}CHCH_2C(O)O_2$ (center) and E-$CH_2(OOH)CH{=}CHCH_2C(O)O_2$ (right). Bond lengths for covalent bond formed or broken in the reactions are are written out in unit Å. k_{MCTST} is the MCTST rate coefficient (Eq. 1 in the main text), κ is the tunneling correction. The subscripts $r0$, ts, $rirc$ and $pirc$ refer to the global minimum reactant, transition state, IRC reactant, and IRC product, respectively.

S5 In search of a TS for CO_2 elimination

As discussed in the main text, out hypothesis is that CO_2 elimination to be the most likely atmospheric fate of highly stained cyclic perester formed from ring closure in β-unsaturated $RC(O)O_2$. There are two possible mechanisms for this: In the first, the elimination occurs directly from the carbon-centered radical formed in the ring closure, in which case the transition state might be stabilized by radical delocalization. In the second, the CO_2 elimination is outcompeted by O_2 addition to the carbon-centered radical, but the excess energy from said addition reaction allows the CO_2 elimination to occur despite the lack of radical delocalization.

In order to search for representative TS geometries for the CO_2 elimination, we started by mapping out the PES leading to the elimination for the acryl peroxy radical, as it is the smallest possible β-unsaturated $RC(O)O_2$. This system may however not perfectly represent the radicals for which this reaction occurs in the atmosphere, since both its ring closure reactions are uncompetitive at tropospheric temperatures (See Table 5 in the main text). Nevertheless, the potential energy surface is presented in Figure S4 at both the DLPNO//jun and F12//aug levels of theory. As expected, the CO_2 elimination is highly exothermic. Surprisingly however, it does not seem to be significantly sped up by entropy in atmospheric conditions. The ring closure TS, the cyclic perester, and the CO_2 elimination TS all have $kT \ln Q$ values within 0.15 kJ/mol of each other at 298 K. This means that TS energy is all-important for determining the viability of the reaction. Unfortunately, it seems that finding an accurate value for this energy will be difficult. As seen in Figure S4, our two CCSD(T) methods disagree on the barrier height by 30 kJ/mol, which is a clear indication that neither of the values is particularly reliable. If T1 diagnostics are any indication, the DLPNO-CCSD(T) value might be slightly more reliable, as on this level of theory the ring closure and CO_2 elimination transition states have similar T1 diagnostics (0.0301 for the former and 0.0345 for the latter), whereas on the RI-CCSD(T)-F12 level of theory the CO_2 elimination TS has a clearly higher diagnostic (0.0353 vs 0.0722). In any case, these results cast some doubt on how well CCSD(T) methods are able to characterize this system at all.

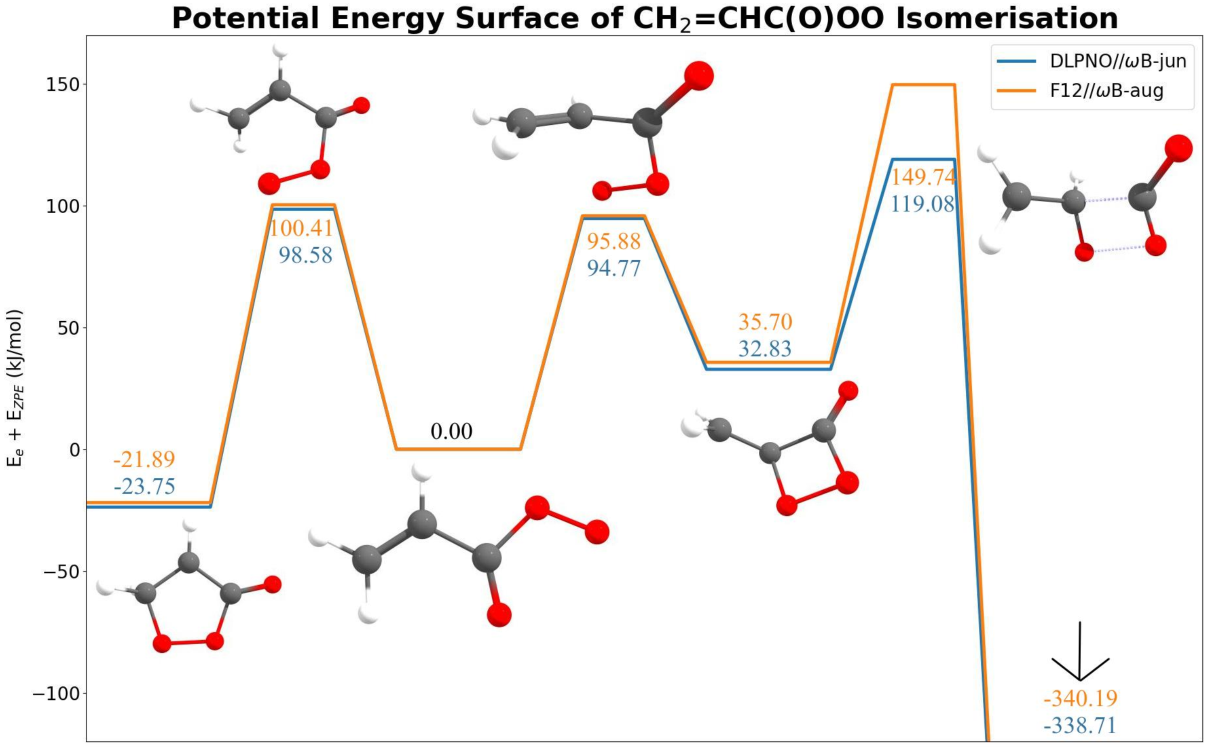

Figure S4: Potential energy surface for ring-closure and CO_2 elimination in the acryl peroxy radical model system.

If we were to trust the DLPNO//jun PES, in which the CO_2 elimination barrier height is 86.25 kJ/mol and, crucially, 24.31 kJ/mol above the ring closure TS in energy, would indicate that the CO_2 elimination is not competitive relative to the ring opening reforming the acryl peroxy radical, let alone O_2 addition. On the other hand, these energetics may be different for β-unsaturated $RC(O)O_2$ with competitive ring closure reactions. As such, we also tried to optimize the CO_2 elimination TS for the fully methyl-substituted $(CH_3)_2C{=}C(CH_3)C(O)O_2$. Unfortunately, we failed to locate a CO_2 elimination TS despite our best efforts, meaning the following: A constrained optimization followed by a saddle point optimization with Hessian calculations at every step, both using B3LYP-D3/ma-def2-SVP, was performed starting from the C-C and O-O bond lengths 1.645 Å and 1.800 Å, as these were the respective bond lengths in the CO_2 elimination TS for $CH_2{=}CHC(O)O_2$. Once this failed, the same was attempted using ωB97X-D3/jun-cc-pVTZ directly. Once this failed as well, a 2D relaxed scan of both bond lengths was performed to locate the approximate region of the TS, whose results are shown in Figure S5. Based on this, a final ωB97X-D3/jun-cc-pVTZ saddle point optimization with Hessian calculations at every step starting from the C-C and O-O bond lengths 1.78 Å and 1.67 Å was performed unsuccessfully.

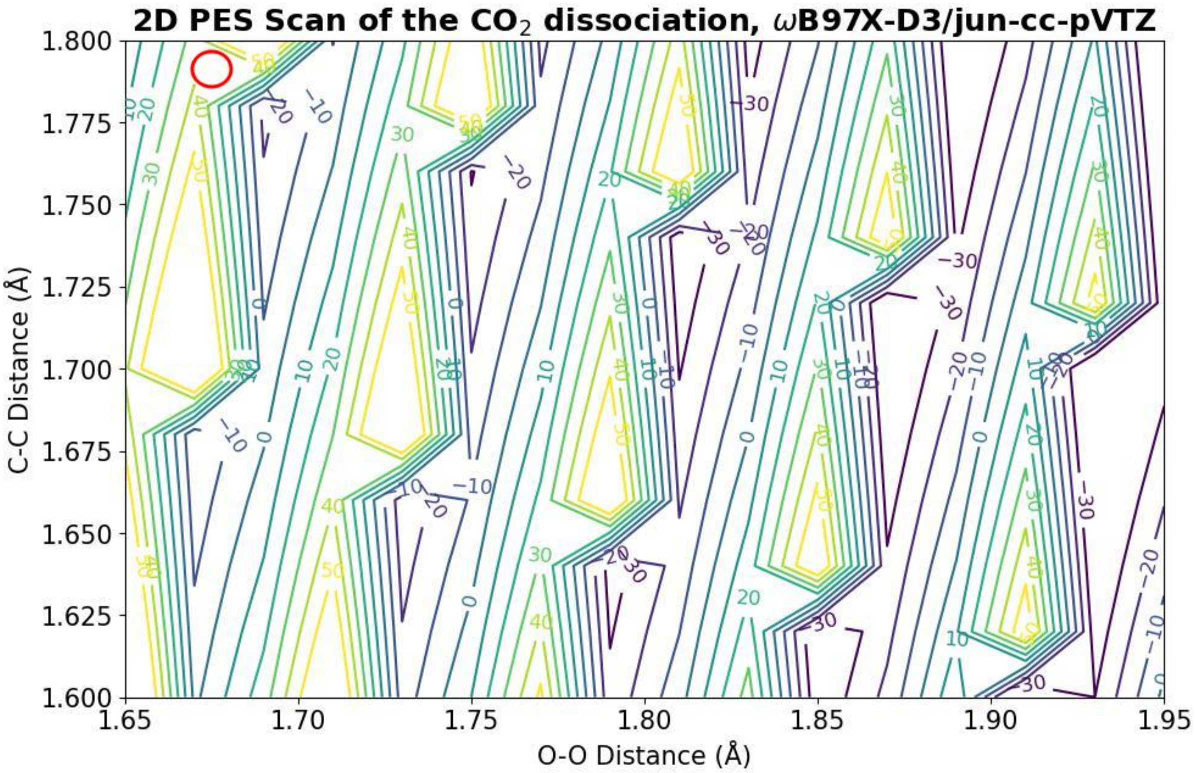

Figure S5: 2D Relaxed scan of the C-C and O-O bond lengths leading to the CO_2 elimination in $(CH_3)_2C{=}C(CH_3)C(O)O_2$. Energies are expressed in kJ/mol relative to the electronic energy of the cyclic perester product of the 4-ring closure, received from ωB97X-D3/jun-cc-pVTZ optimization on top of the IRC product structure. The apparent location of the TS is marked with a red circle.

Nevertheless, from the results of the 2D relaxed scan (Figure S5) we see that the PES around the CO_2 elimination is full of tiny hills and valleys, which are likely the cause behind the difficulties in the saddle point optimizations. Whether these hills and valleys are true physical properties or an artifact of our density functionals' mischaracterization of an evidently multi-configurational reaction can not be determined without multi-reference methods. If the energetics predicted by the ωB97X-D3/jun-cc-pVTZ methods are to be trusted, however, the TS saddle point would be approximately 40 kJ/mol above the cyclic perester in electronic energy. This is a stark contrast to $CH_2{=}CHC(O)O_2$, where the corresponding difference on the ωB97X-D3/jun-cc-pVTZ PES was 90.57 kJ/mol. This offers some indication that the CO_2 elimination might in fact be more competitive for the more substituted β-unsaturated $RC(O)O_2$, but the results are highly uncertain, as it is difficult to tell how reliable any of these values are. In conclusion then, we are neither able to confirm nor eliminate the occurrence of CO_2 elimination following 4-ring closure in β-unsaturated $RC(O)O_2$. We can only conclude that these reactions are possible.

References

[1] L. Vereecken and B. Nozière, H migration in peroxy radicals under atmospheric conditions, *Atmos. Chem. Phys.*, 2020, **20**, 7429–7458.

[2] P. Seal, S. Barua, S. Iyer, A. Kumar and M. Rissanen, A systematic study on the kinetics of H-shift reactions in pristine acyl peroxy radicals, *Phys. Chem. Chem. Phys.*, 2023, **25**, 28205–28212.

[3] G. da Silva, Hydroxyl radical regeneration in the photochemical oxidation of glyoxal: kinetics

and mechanism of the HC(O)CO + O_2 reaction, *Phys. Chem. Chem. Phys.*, 2010, **12**, 6698–6705.

[4] M. P. Rissanen, T. Kurtén, M. Sipilä, J. A. Thornton, J. Kangasluoma, N. Sarnela, H. Junninen, S. Jørgensen, S. Schallhart, M. K. Kajos, R. Taipale, M. Springer, T. F. Mentel, T. Ruuskanen, T. Petäjä, D. R. Worsnop, H. G. Kjaergaard and M. Ehn, The formation of highly oxidized multifunctional products in the ozonolysis of cyclohexene, *J. Am. Chem. Soc.*, 2014, **136**, 15596–15606.

[5] S. Barua, S. Iyer, A. Kumar, P. Seal and M. Rissanen, An aldehyde as a rapid source of secondary aerosol precursors: theoretical and experimental study of hexanal autoxidation, *Atmos. Chem. Phys.*, 2023, **23**, 10517–10532.

[6] K. H. Møller, K. H. Bates and H. G. Kjaergaard, The importance of peroxy radical hydrogen-shift reactions in atmospheric isoprene oxidation, *J. Phys. Chem. A*, 2019, **123**, 920–932.

[7] L. Franzon, M. Camredon, R. Valorso, B. Aumont and T. Kurtén, Ether and ester formation from peroxy radical recombination: a qualitative reaction channel analysis, *Atmos. Chem. Phys.*, 2024, **24**, 11679–11699.

[8] J. D. Crounse, H. C. Knap, K. B. Ørnsø, S. Jørgensen, F. Paulot, H. G. Kjaergaard and P. O. Wennberg, Atmospheric fate of methacrolein. 1. Peroxy radical isomerization following addition of OH and O2, *J. Phys. Chem. A*, 2012, **116**, 5756–5762.

[9] A. P. Teng, J. D. Crounse and P. O. Wennberg, Isoprene peroxy radical dynamics, *J. Am. Chem. Soc.*, 2017, **139**, 5367–5377.

[10] E. Praske, R. V. Otkjær, J. D. Crounse, J. C. Hethcox, B. M. Stoltz, H. G. Kjaergaard and P. O. Wennberg, Atmospheric autoxidation is increasingly important in urban and suburban North America, *Proc. Natl. Acad. Sci.*, 2018, **115**, 64–69.

[11] S. Grimme, Supramolecular binding thermodynamics by dispersion-corrected density functional theory, *Chem. - Eur. J.*, 2012, **18**, 9955–9964.

[12] K. Dill and S. Bromberg, *Molecular driving forces: statistical thermodynamics in biology, chemistry, physics, and nanoscience, 1st Edition*, Garland Science, 2003, pp. 183–185.

[13] P. Pracht, S. Grimme, C. Bannwarth, F. Bohle, S. Ehlert, G. Feldmann, J. Gorges, M. Müller, T. Neudecker, C. Plett *et al.*, CREST—A program for the exploration of low-energy molecular chemical space, *J. Chem. Phys.*, 2024, **160**, year.

[14] P. Pracht and S. Grimme, Calculation of absolute molecular entropies and heat capacities made simple, *Chem. Sci.*, 2021, **12**, 6551–6568.

[15] S. Iyer, A. Kumar, J. Zhao, S. Barua, A. Savolainen, A. Ojala, L. Pichelstorfer, P. Seal, M. Boy, M. Ehn, P. Roldin and M. P. Rissanen, Para-Xylene Oxidation, Manuscript in Preparation.

[16] H. C. Knap and S. Jørgensen, Rapid hydrogen shift reactions in acyl peroxy radicals, *J. Phys. Chem. A*, 2017, **121**, 1470–1479.
